# Factors Affecting Accuracy of Data Abstracted from Medical Records

**DOI:** 10.1371/journal.pone.0138649

**Published:** 2015-10-20

**Authors:** Meredith N. Zozus, Carl Pieper, Constance M. Johnson, Todd R. Johnson, Amy Franklin, Jack Smith, Jiajie Zhang

**Affiliations:** 1 Duke Translational Medicine Institute, Durham, North Carolina, United States of America; 2 Duke University School of Medicine, Durham, North Carolina, United States of America; 3 Duke University School of Nursing, Durham, North Carolina, United States of America; 4 University of Texas, School of Biomedical Informatics, Houston, Texas, United States of America; Liverpool School of Tropical Medicine, UNITED KINGDOM

## Abstract

**Objective:**

Medical record abstraction (MRA) is often cited as a significant source of error in research data, yet MRA methodology has rarely been the subject of investigation. Lack of a common framework has hindered application of the extant literature in practice, and, until now, there were no evidence-based guidelines for ensuring data quality in MRA. We aimed to identify the factors affecting the accuracy of data abstracted from medical records and to generate a framework for data quality assurance and control in MRA.

**Methods:**

Candidate factors were identified from published reports of MRA. Content validity of the top candidate factors was assessed via a four-round two-group Delphi process with expert abstractors with experience in clinical research, registries, and quality improvement. The resulting coded factors were categorized into a control theory-based framework of MRA. Coverage of the framework was evaluated using the recent published literature.

**Results:**

Analysis of the identified articles yielded 292 unique factors that affect the accuracy of abstracted data. Delphi processes overall refuted three of the top factors identified from the literature based on importance and five based on reliability (six total factors refuted). Four new factors were identified by the Delphi. The generated framework demonstrated comprehensive coverage. Significant underreporting of MRA methodology in recent studies was discovered.

**Conclusion:**

The framework generated from this research provides a guide for planning data quality assurance and control for studies using MRA. The large number and variability of factors indicate that while prospective quality assurance likely increases the accuracy of abstracted data, monitoring the accuracy during the abstraction process is also required. Recent studies reporting research results based on MRA rarely reported data quality assurance or control measures, and even less frequently reported data quality metrics with research results. Given the demonstrated variability, these methods and measures should be reported with research results.

## Introduction

Data have been abstracted from medical records since the earliest days of medical record keeping.[1] Unfortunately, the medical record abstraction (MRA) process remains largely uncharacterized and produces inconsistent results.[2]

MRA, also referred to as chart review, is a common method of data collection for research and secondary data use[3–6]—for example, reviews of studies in emergency medicine and nursing report that 25% to 53% of the reviewed articles relied on abstracted data.[5–7]

Although many studies today can be conducted with electronically extracted data, smaller studies often do not have the resources to program and validate extraction routines or apply natural language processing to narrative data. Thus, progress in electronic medical record adoption and increasing use of electronically extracted data do not obviate the need for MRA. Even with electronic medical records, abstractors often still page through screen by screen to identify the data values for abstraction and remain hampered by many of the same issues affecting abstraction from paper charts. Further, MRA is a primary method for validating phenotypes for electronically extracting data from healthcare information systems.[8] Such validation remains subject to the same MRA quality challenges. Thus, for the foreseeable future, data accuracy from MRA remains a concern.

Hospital administrators ranked MRA systems highest among common information systems and sources for data accuracy.[9] However, many others have questioned the adequacy of data abstracted from medical records to support clinical research. As early as 1969, MRA was associated with poorly described processes and with inconsistency and error.[10] In a recent review, MRA was associated with a median error rate that was an order of magnitude higher than other data collection and processing methods.[2] Recent publications have highlighted the persistence of data accuracy problems, including high and highly variable error rates of sufficient magnitude to cause problems in analysis of abstracted data.[11–13]

Although concern about quality of data abstracted for secondary use from medical records dates back at least to 1746,[1] factors having an impact on data accuracy have not been systematically analyzed or described. The literature contains several hundred articles mentioning MRA problems or abstraction methods; however, existing work is mostly observational in nature and lacks even an authoritative definition of MRA. According to Meads and Cooney, because the medical record is the traditional source for clinical information, its use for non-clinical purposes has been largely unquestioned.[14] For example, in clinical trials using abstracted data, clinical trial monitors routinely compare at least a percentage of the data on the collection form to the medical record in a process called source data verification. However, MRA data error rates are usually not measured, nor are they presented with the analysis. Likewise, MRA error rates are rarely reported in secondary analysis studies. Lowenstein recently maintained that Feinstein et al.’s 1969 intimation remained true: “medical record reviews are still governed by the ‘laws of laissez faire: the investigator usually chooses the records and removes the data in whatever manner he wishes, and he seldom reports the details of this method.”‘[10,15] Further, a recent article reported that although the authors “tried to conduct data abstraction according to the best advice found in the literature,” they nonetheless encountered challenges not described in the literature.[16]

**Fig 1:**
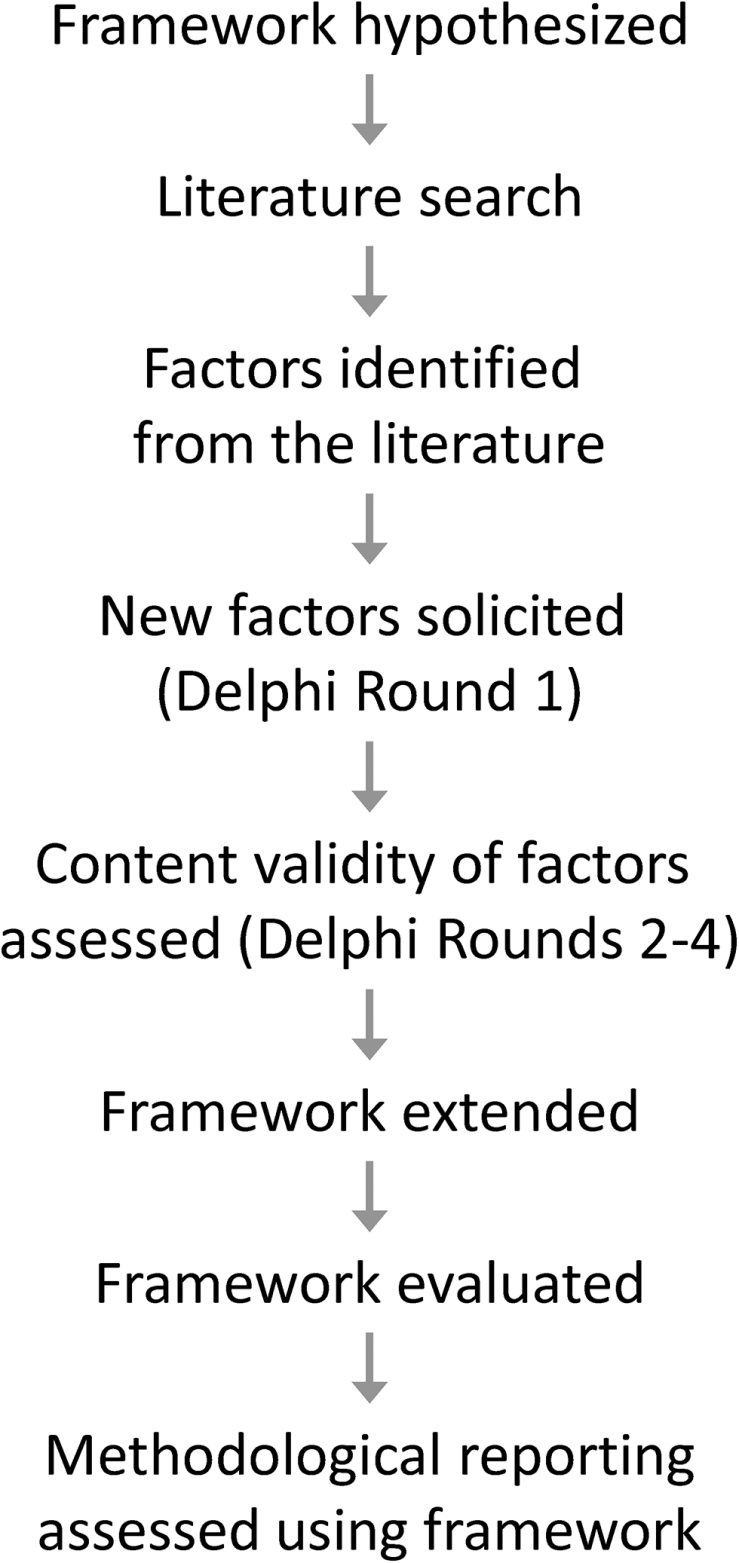
Overview of the research process.

The objectives of our research were 1) to identify and validate the factors affecting the accuracy of data abstracted from medical records and 2) to combine the factors in an engineering control theory based framework for data quality assurance and control in medical record abstraction. The process followed to meet these objectives is depicted in Fig 1 and described below.

## Materials and Methods

### 2.1 Definitions

Since an authoritative definition of MRA has not yet been articulated, the following operational definition of MRA was developed using concept analysis methodology and personal experience. For this research, we define MRA as a process in which a human manually searches through an electronic or paper medical record to identify data required for a secondary use. Abstraction involves some direct matching of information found in the record to the data required, and commonly includes operations on the data such as categorizing, coding, transforming, interpreting, summarizing, or calculating. The abstraction process results in a summary of information about a patient for a specific secondary data use.

### 2.2 Literature Search

**Fig 2:**
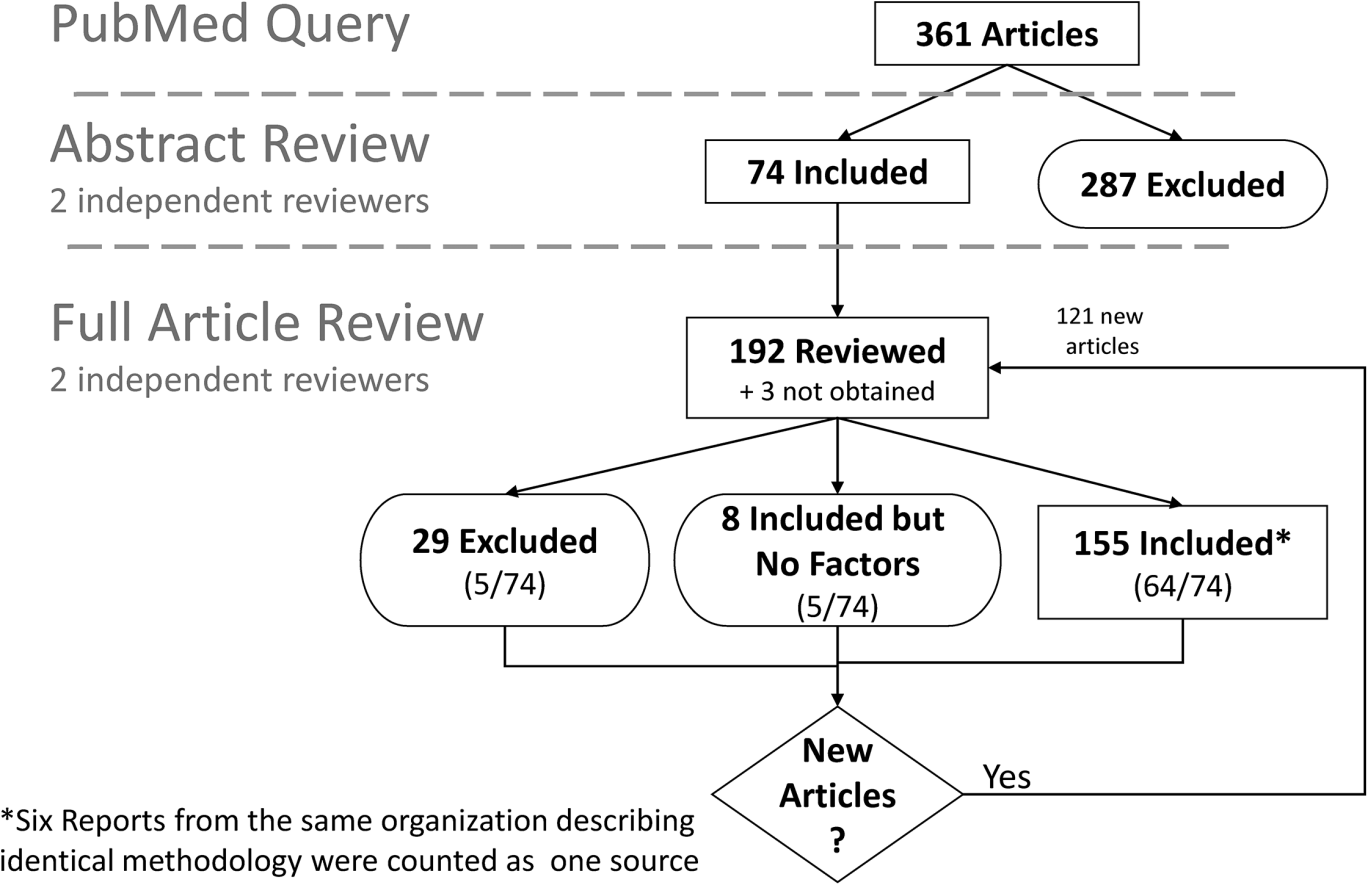
Disposition of screened articles.

A literature search was conducted in PubMed (S1 Appendix) in October 2009. The PubMed search identified 361 articles, and a review of reference lists identified an additional 121. Of the total of 482 potentially relevant citations, 192 were reviewed in full text, and 155 of those were retained for analysis (Fig 2). Abstracts and articles were screened by two independent reviewers, and disagreements were resolved by discussion.

### 2.3 Inclusion Criteria

Articles were retained for analysis based on the following inclusion criteria: 1) articles in the English language, 2) articles describing use of healthcare data with the medical record as the source (later clarified as healthcare of humans), and 3) reports of, perspectives on, case studies using, or results of MRA. The list of included articles is provided in S2 Appendix.

### 2.4 Data Collection

The full text of the 155 included articles was reviewed to identify and name factors contributing to the quality of data abstracted from medical records. In this first-pass review, categories of factors were created by grouping similar concepts as the articles were read generating an initial set of factor names (i.e., codes). These codes were entered into NVivo qualitative analysis software (QSR International, Victoria, Australia) for use during the double independent coding of the included articles and, later, the Delphi Round 1 results.

Following the first pass, two independent coders read the full articles and marked each sentence, mentioning anything affecting (increasing, decreasing, or stated without valence) the accuracy of data abstracted from medical records. Sentences identified by either coder were entered into NVivo for coding. Each of the coders had a master’s degree and experience in clinical research, registries, and healthcare quality improvement (QI) projects.

The two coders accessed the excerpted sentences in NVivo and coded anything stated or implied as affecting the accuracy of data obtained through MRA. Each reviewer was instructed to code semantically similar factors to the same code—for example, “re-abstraction” and “re-review of charts” were coded to and counted as one factor rather than two distinct factors. Factors stated at different levels of granularity or with different modifiers or context (e.g., “re-abstraction” vs. “ongoing re-abstraction”) were retained as separate factors. In the example, re-abstraction is repeated abstraction, while ongoing re-abstraction has the added context of occurring throughout a project, and thus the two are different concepts. Similarly, factors stated with opposing valence were retained as distinct factors (e.g., “training abstractors increases accuracy” vs. “lack of training decreases accuracy”). New codes were added as new concepts were encountered. The coded factors from each coder were compared, and disagreements were resolved through discussion between the two coders to arrive at the set of factors identified from the literature.

### 2.5 Ethics Statement

Approval was received from the Duke University (Pro00019240) and University of Texas (HSC-SHIS-09-0367) institutional review boards for the following Delphi portion of the study. Written consent was obtained from all research participants.

### 2.6 Assessing Factor Validity via Delphi Processes

Content validity of the top 75 factors reported in the literature was assessed through two separate Delphi processes.[17] Experienced medical record abstractors were recruited for each Delphi process. The first Delphi process recruited clinical research abstractors and the second recruited registry and QI abstractors. Clinical research abstractors were recruited at the Society for Clinical Research Associates national conference in September 2009. Registry and QI abstractors were recruited at the American Health Information Management Association national convention in September 2009. Eligible participants were individuals having 3 or more years of abstraction experience as reported by the participant, abstraction experience in either a clinical research or registry/QI setting, and ability and willingness to give informed consent. Twenty clinical research and 18 registry/QI abstractors were ultimately consented to participate in this study to ensure a minimum of seven participants remaining at the end of the last Delphi rounds.

In addition to assessing content validity of the factors synthesized from the literature, the Delphi processes were used to better understand the importance and reliability of the factors identified from the literature and to identify differences between clinical research and registry/QI abstractors in factors perceived as impacting the accuracy of abstracted data. A four-round Delphi process was used for both the clinical research abstractors and the registry/QI abstractors. Delphi Rounds 1 and 2 were conducted using the Cogix web-based survey system supported by the Duke Translational Medicine Institute. Rounds 3 and 4 were conducted via structured phone interview. Member checking occurred as part of the Delphi design, in which participants saw the aggregate results of each previous Delphi round. A peer debriefing session was conducted with 30 independent study coordinators from Duke University Medical Center in February 2010.

1. In Delphi Round 1, participants were asked to list from five to 10 factors that, based on their experience, affected the accuracy of abstracted data. This open-ended question approach was used in Round 1 to prevent biasing the participants with the factors we synthesized from the literature and to measure the number of participant-reported factors that were not found in the literature. Thus, Round 1 of the Delphi retained the potential to identify new factors not reported in the literature. Following Round 1, the factors reported by participants were reviewed and coded to obtain a list of distinct Delphi Round 1 factors.
2. For Round 2, the top 75 factors identified in our literature search were combined with those obtained from Delphi Round 1 (disposition of factors throughout the process is detailed in S3 Appendix and S1 Fig). The coded factors were grouped into categories via card sorting by the first author. Definitions of each factor were provided to ensure consistent expression of similar concepts. Each factor was presented as a statement that the factor either increased or decreased the accuracy of abstracted data (e.g., “Training abstractors on data collection forms increases the accuracy of abstracted data”). The participants were asked to rate their level of agreement with each statement on a 5-point Likert scale (strongly disagree, mildly disagree, neither agree nor disagree, mildly agree, or strongly agree).
3. In Round 3, the participants were each provided an individualized report of their Round 2 responses versus the aggregate responses and interviewed over the telephone. To prevent bias, an independent interviewer (i.e., not one of the investigators) was used. In the Round 3 interviews, each participant was asked for more information about factors where their response was within 1 point of the aggregate and factors where their response differed by more than 1 point from the aggregate. One point was chosen because a difference of 1 point is the difference between the categories on the Likert scale. Participants were permitted to change their responses if they wished to do so. Interviewing participants about their responses enabled researchers to clarify responses and to assess consistency of understanding of the statements on the questionnaire. The Round 3 interviews also provided the researchers more in-depth information about factors for which participants’ answers depended on things external to the stated factor such as differences in types of trials, types of registries, types of QI projects, or clinical area.
4. In Round 4, the participants were each provided an individualized report of their Round 3 responses and the aggregate responses. Participants were again interviewed and permitted to change their responses. In an attempt to better understand disagreements in ratings identified in Rounds 2 and 3, participants were also asked for more information about factors where their response was within 1 point of the aggregate and factors where their responses differed by more than 1 point from the aggregate.

### 2.7 Analysis

Content validity of the factors synthesized from the literature was assessed in two ways: 1) completeness as measured by the number of new factors stated in Round 1 of the Delphi ultimately rated as important (i.e., mildly agree or strongly agree) in Round 4, and 2) consistency between the factors identified in our literature search and the Delphi expert panels. Finally, interclass correlation (ICC) analysis was used to assess reliability of the factors according to the Delphi ratings. Cutoffs were established for factor importance and factor reliability, and factors judged as not important or not reliable were not included in the resulting framework. Data were analyzed using SAS software (SAS, Cary NC, USA).

### 2.8 Framework Development

**Fig 3:**
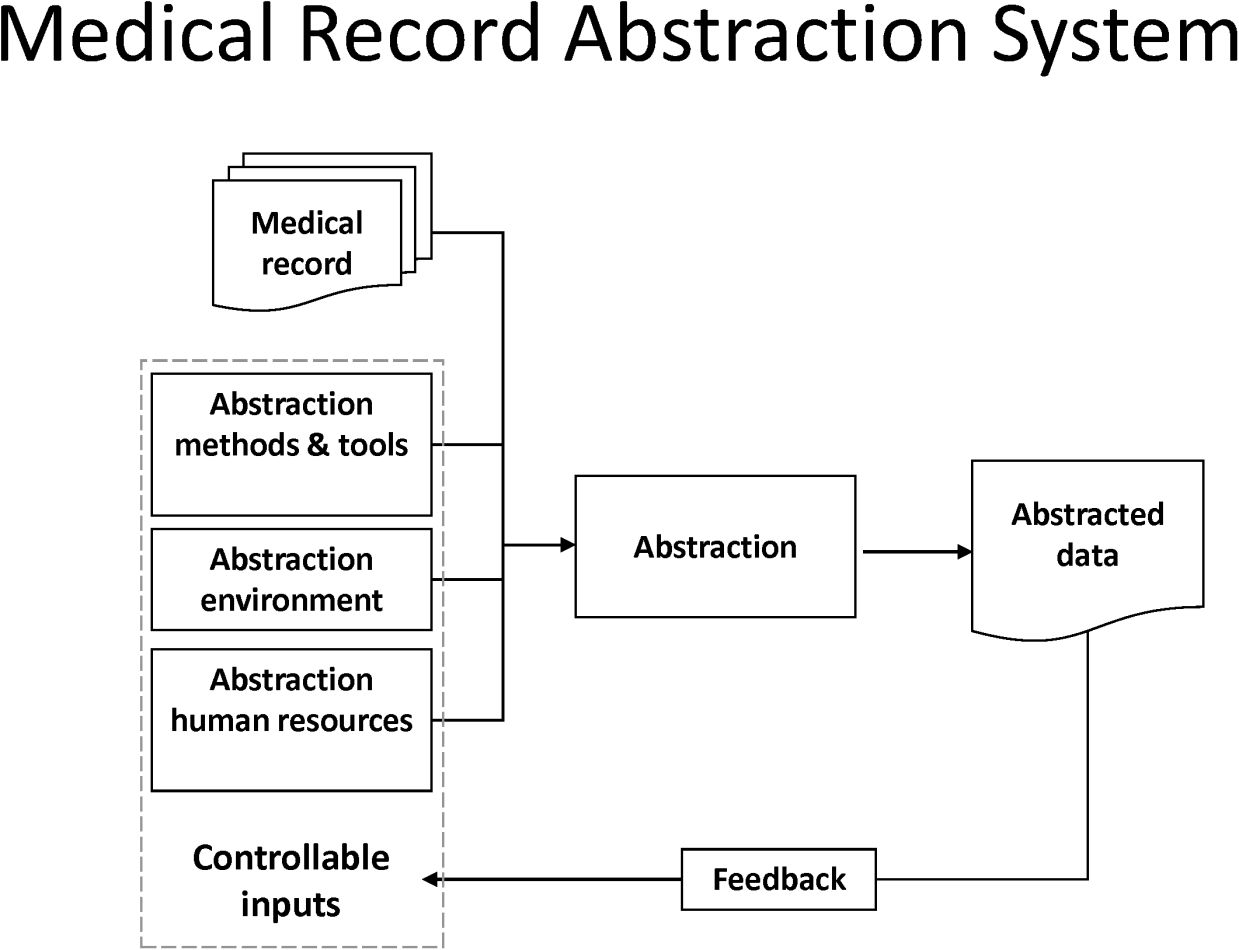
The MRA system.

A high-level framework (Fig 3) was deductively hypothesized from engineering control theory in preparation for this research. We posited that the MRA process could be described as a system (i.e., a process) that converts inputs to outputs, with the output being the abstracted data. Further, as a system, we posited that feedback is obtainable and that the feedback can be used to help control or improve the process. We postulated that input factors may be controllable or non-controllable. We asserted four mutually exclusive categories of input factors that might affect the quality of data abstracted from medical records: 1) the medical record (not controllable by secondary data users), 2) abstraction methods and tools, 3) abstraction environment, and 4) abstraction human resources. We considered categories 2–4 to be often controllable by secondary data users. The dotted line used for the box around the controllable inputs in Fig 3 signifies that for some organizations or studies, factors may be controllable in one case but not controllable in another. The feedback loop represents feedback to the abstractor and abstraction process, such as from a re-abstraction type review. In keeping with control theory, this feedback can be used to improve the accuracy of abstracted data—that is, to influence controllable factors or mitigate the impact of non-controllable factors to increase the quality of the output (abstracted data). The initial framework was purposefully high-level and not imposed on the factor coding. After the Delphi and ICC analysis, the individual validated factors were grouped under the four high-level categories as described in S3 Appendix, to provide detail at an actionable level for investigators and research teams.

### 2.9 Framework Evaluation

The framework was evaluated using articles published in 2011 and 2012. The framework was evaluated based on content coverage—that is, the extent to which factors mentioned in the 2011–2012 literature were accounted for in the framework. Content coverage was operationalized as the percentage of factors identified in the literature that were an exact match with or contained within the framework factors.

We also evaluated the comprehensiveness of methodological reporting in the current literature. Comprehensiveness of methodological reporting was operationalized as presence of any described activity from each of the four highest-level framework domain areas: 1) data source within the medical record identified, 2) abstraction methods and tools stated, 3) abstraction environment described, and 4) abstraction human resources described. A mention of any factor falling within a category was sufficient to score the article positive in that category. For example, an article reporting the specific data source as “procedure reports in the patient’s medical record” would score the article positive for category 1 above. Similarly, articles were also scored for specific mention of quality assurance or control activities.

The PubMed search strategy used for the initial literature review was used to identify articles for the framework evaluation (S1 Appendix). A total of 104 articles were retrieved and screened according to the criterion used in the initial literature search, resulting in 66 full text articles retained for evaluation.

## Results

### 3.1 Factors Identified from the Literature

We identified a total of 2385 factors impacting the accuracy of data abstracted from medical records. Of these, there were 292 distinct factors.

### 3.2 Factors Identified from Round 1 of the Delphi

The net result after the first Delphi round was that four new semantically distinct factors were identified. Round 1 of the Delphi identified 227 total factors. Six of the 227 items fell outside of the working definition of MRA, and five items could not be classified due to ambiguity of the information provided by the participant, leaving 216 factors. Of these 216, 92 were distinct. Twenty-five (27%) of the distinct factors were stated by more than two participants.

Four factors identified in Round 1 were not mentioned at all in the articles included in the literature review. Five factors were not complete semantic matches at the detail level at which they were mentioned but were conceptually part of higher-level factors or were related to factors mentioned in the literature (Table 1). For example, the concept of abstractor credentials was identified 10 times during the Delphi process, while in the literature, the concept of credentials was described variously as “necessity of a registered nurse (RN),” “presence of an advanced degree,” and “certification of abstractors.”[18–21] Factors such as these at different levels of granularity were not combined for counting purposes; however, to aid reproducibility of this research, they are listed in Table 1. Table 2 lists factors identified by Delphi Round 1 that were also identified in the literature review but were not in the top 26% of the literature-identified factors.

**Table 1:** Factors identified in Delphi Round 1 that were not in the literature.

Factor	Number of mentions
Abstractor credentials^*^	10
Access to charts^*^	6
Interruptions^†^	6
Complete and accurate medical record^*^	4
Availability of abstraction tools^*^	4
Adequate time for abstraction tasks^*^	4
Complexity of the study or project^†^ ^,^ ^‡^	3
Supportive collegial relationships with physicians, nurses and medical records colleagues^†^	3
Abstractor (human) error^†^	3

^*^ Not complete semantic matches at the detail level at which they were mentioned but conceptually part of higher-level factors or related to factors mentioned in the literature.

^†^ Not mentioned at all in the articles included in the systematic review.

^‡^ Ultimately not upheld in Delphi Round 4.

**Table 2:** Factors identified in Delphi Round 1 that were not in the literature top 26%.

Factor	Number of mentions in Delphi / literature
Limited time	5 / 1
Lack of training^*^	4 / 2
Same information found in multiple places in the medical record (opportunity for conflicting information)	3 / 3
Incomplete review of the medical record	3 / 1
Volume of information in the medical record^†^	3 / 1

^*^ Found in the literature top 26% but with opposite valence.

^†^ Ultimately not upheld in Delphi Round 4.

### 3.3 Factors Carried Forward into Delphi Round 2

Combining the 75 top literature factors (top 26%, those found in more than three articles in the literature search) and the 25 top Delphi Round 1 factors (top 27%, those identified by more than two Delphi participants) provided 100 total factors, 89 of them distinct (Table 3). Only these top factors could be carried forward in Round 2 because the participants were consented for a 1-hour or shorter time commitment per Delphi round.

**Table 3:** Comparison of factors mentioned in the Delphi top 27% and the literature top 26%.

	Literature top 26%		
Delphi top 27%	Mentioned	Not mentioned	Total
Mentioned	11	14	25
Not mentioned	64	_	64
Total	75	14	89

The questionnaire used in Delphi Round 2 was created using these 89 distinct factors, as described in S3 Appendix. After conflated concepts were split, like concepts were combined, and the 14 categories were expanded for exhaustiveness and mutual exclusivity a total of 99 factors carried forward into Delphi Round 2.

### 3.4 Content Validity Assessment

**Fig 4:**
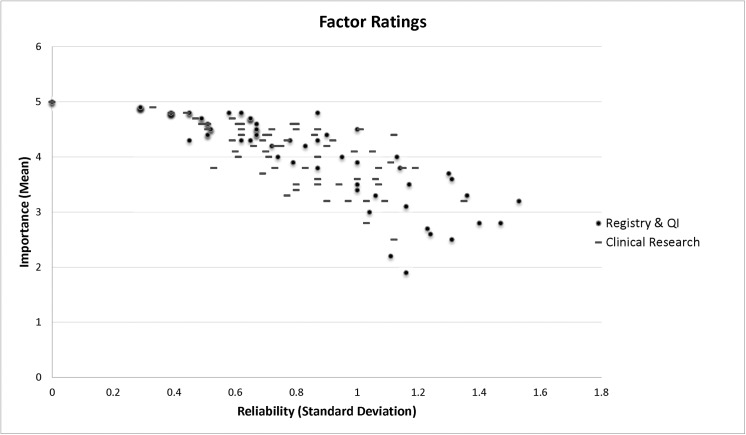
Importance versus reliability of factors.

Content validity was assessed after the fourth (last) Delphi round. Combining both Delphis (i.e., the clinical research and the registry/QI Delphis), there were 77 factors (78.8%) with overall average ratings between mildly and strongly agree, 78 (78.5%) registry and QI, and 75 (75.8%) clinical research. We analyzed importance (mean) versus the reliability or stability (standard deviation [SD]) of the ratings for each factor (Fig 4), the ideal combination being high importance and low variance in the ratings (i.e., top left corner of the graph). The more important the factor, the more consistently it was rated by both Delphi panels.

We drew a cutoff at SD > 1.2 (slightly greater than the distance between two points of the Likert scale) and a second at mean < 3 (neutral). Factors rated lower than neutral were considered to be refuted by the Delphi processes. Factors with a rating SD > 1.2 were considered to be suspect due to lower reliability of the ratings.

Three factors had an overall rating lower than neutral (Table 4); all of these were rated between mildly disagree and neutral. The registry and QI Delphi rated seven factors lower than neutral (Table 4). The clinical research Delphi rated two factors lower than neutral. All but one factor rated lower than neutral (Table 4) originated from the literature.

**Table 4:** Refuted and uncertain factors.

Refuted Factor	Clinical research mean (σ)	Registry / QI mean (σ)	Overall mean (σ)	Number of comments (%) mentioning mitigating factors
Necessity of RN credential	3.2 **(1.35)**	**2.2** (1.11)	**2.8 (1.35)**	13 (62%)
Blinding abstractors to study aims	**2.5** (1.12)	**1.9** (1.16)	**2.2** (1.15)	10 (20%)
Centralized abstraction	3.2 (0.97)	**2.7 (1.23)**	3.0 (1.1)	8 (63%)
High study/project complexity	3.8 (1.07)	**2.5 (1.31)**	3.3 **(1.33)**	11 (64%)
High volume of data in medical records^*^	3.2 (1.09)	**2.8 (1.47)**	3.1 **(1.25)**	14 (29%)
Care provided by multiple providers/facilities^*^	3.6 (1.06)	**2.8 (1.40)**	3.3 **(1.25)**	12 (33%)
Presence of multiple diagnoses/procedures	**2.8** (1.03)	**2.6 (1.24)**	**2.7 (1.11)**	8 (50%)
Abstractors with different levels of experience^*^	3.2 (1.03)	3.6 **(1.31)**	3.4 (1.15)	16 (44%)
Abstracting from narrative text^*^	4.2 (0.90)	3.3 **(1.36)**	3.8 (1.2)	6 (50%)
Coding data while abstracting	3.3 (0.77)	3.2 **(1.53)**	3.2 (1.12)	10 (30%)
Same information found in multiple places	3.9 (1.11)	3.7 **(1.30)**	3.8 (1.18)	8 (25%)

Marked mean values in the table are those rated lower than neutral. Marked standard deviation values in the table are those that were above the standard deviation of 1.2 cut-off.

^*^ Comments mentioning a mitigating factor as well as justification for participants’ response were split into two. The factor “Abstractors with different levels of experience” had two comments split; remaining marked factors had one comment split.

Abbreviations: QI, quality improvement; RN, registered nurse.

Factors with an SD > 1.2 were explored with participants to the extent possible within the time constraints of the Round 3 interviews. Pertinent verbatim statements from the interviews are included in S4 Appendix. The numbers (and percentages) of comments that mentioned other factors which were more important or mitigated the impact of the factor in question are displayed in Table 4.

In summary, the four factors exhibiting low reliability (Table 4) were dropped from the list of factors. A total, of 11 (11%) of the 99 Delphi vetted factors were not upheld by the Delphi, i.e., dropped for low importance or low reliability. The ICC, looking at the variance of the question ratings and controlling for rater variance (person) and error variance (person by question), was 0.343. The ICC for the clinical research Delphi was 0.298, and the ICC for the registry Delphi was 0.457. The ICC results show that there was greater variability in the registry and QI Delphi. The Delphi processes resulted in 88 verified factors.

### 3.5 Framework Extension

The 88 Delphi-vetted factors were used to extend the high-level framework (Fig 3). The 204 (292–88) untested factors were not included in the framework. The 88 factors were further consolidated as described in S3 Appendix by removing six factors of opposite valence, and lumping two sets of factors with closely related factors (noted in Table 5).

**Table 5:** Framework for increasing data accuracy in MRA.

**Abstractor Human Resources**
Abstractor qualification
•Abstractor familiarity with how data are recorded in the medical record
•Abstractor experience in the clinical area for which he or she is abstracting
•Abstractor experience abstracting in any clinical area
•Abstractor having a clinical credential in the area in which he or she is abstracting
•Abstractor having passed a competency test
Communication with abstractors
•Providing feedback to abstractors, e.g., from periodic review of cases
•Ongoing communication with abstractors, e.g., opportunity to discuss difficult cases
Abstractor project specific training
•Training abstractors^*^
•Ongoing abstractor training
•Recommended components of training
a.) Instruction in the therapeutic area, i.e., clinical specialty, of the study or registry
b.) Instruction in how data are collected in the healthcare setting or specifically at participating facilities
c.) Including an overview of the study or registry, e.g., purpose, how the data will be used
d.) Covering abstraction specifications, i.e., data element definitions, guidelines, and conventions
e.) Training abstractors on the data collection or abstraction form
f.) Training on proper use of coding systems used^†^
g.) Covering the software to be used in the abstraction, i.e., computerized abstraction form
h.) Practice exercises with feedback
i.) Examples of difficult cases
**Abstracting Environment**
•A supportive and positive relationship with local physicians, nurses, and medical records colleagues
•Minimizing interruptions during abstraction
•Minimizing time pressure, i.e., limited time in which to abstract
•Easy access to medical records
•Facilities having training for clinicians in better documentation practices
**Abstraction Methods and Tools**
Abstraction process
•Abstracting data during the patient encounter, i.e., while the patient is in the hospital
•Using data collection forms, i.e., abstraction forms
•Conducting a pilot study of the abstraction
•Reviewing the entire or relevant parts/time period of the medical record before abstracting^*^
Applying methods that decrease human error^*^ ^,^ ^‡^
•Availability of abstraction tools, e.g., guidelines, conventions, definitions
Abstraction guidelines
•Standardizing the abstraction process by explicit criteria, i.e., rules or conventions for each data element
•Specifying the location in the medical record where the data element is to be found^*^
•Prioritizing the locations in the medical record where data elements may be found
•Documenting inclusion and exclusion criterion defining which cases should be in the study or registry
•Documenting rules for dealing with missing information
•An available glossary with synonyms and abbreviations
Data element definition
•Defining each data element
•Specifying categories to denote unknown information, i.e., null flavors
•Denoting and prioritizing critical data elements
•Providing conventions describing handling of common problems, e.g., multiple values, missing information, for each data element
•Valid values for categorical data elements, i.e., data elements with check boxes or pick lists, should cover all possible options and not overlap
•Choosing valid values that resolve minor discrepancies, i.e., broad categories
•Defining and collecting data elements as structured data rather than free text
•Defining data elements as raw data, i.e., data that are abstracted directly from medical records rather than those requiring mapping, interpretation, calculations, converting units, or scoring questionnaires^§^
•Assuring that data elements are routinely documented in medical records, i.e., documented in standard care
•Avoiding subjective data elements, i.e., those requiring judgment, or constraining the subjectivity through definition to make the data element more objective, i.e., more likely to be abstracted consistently
•Identifying data elements abstracted less accurately, e.g., pain onset time, symptoms, procedure onset time, and constraining them through definition, guidelines, or examples, or avoiding them altogether
•Using data elements that are the original recording rather than those that may be transcribed from other places, e.g., using original lab values from the lab rather than those transcribed into a discharge summary
Data collection or abstraction form
•Using a well-designed data collection form
•Using the same form, i.e., a standard form, at multiple data collection sites
•Listing codes on the data collection form for data elements where the abstractor assigns a code during abstraction
•Ordering questions on the data collection form following the order in the medical record
Computer use in abstraction
•Entering data into a computer as the data are abstracted
•Use of computerized error checks during data entry to notify the abstractor about missing, out-of-range, or illogical values
•Use of computerized error checks after data entry for missing, out-of-range, or illogical values
•Minimizing transcription steps, e.g., transcribing data from the data collection form to the database
•Use of independent data sources to verify data, e.g., checking data against another source of the same data
**Factors Inherent in Medical Records that Decrease the Accuracy of Abstracted Data**
•Error and inconsistency in the medical record^*^
•The practice of providers not documenting results or assessments that are “normal,” i.e., only charting pertinent negatives
•Missing information, i.e., incompleteness of the medical record^*^
•Missing charts, i.e., instances when the medical record is not available
•Conflicting information in the medical record
•Illegible information in the medical record
•Uncertainty in the medical record, i.e., statements such as “possible infarction” rather than a firm diagnosis
•Variability of documentation practices among clinicians
•Variability of assessment skills, i.e., of the clinician examining the patient
**Data Quality Control Activities**
Aspects of re-abstraction
•Re-abstraction of data
•Reviewing or re-abstracting a representative selection of cases rather than all of the cases
•Independent or external re-abstraction, i.e., re-abstraction by someone other than the initial abstractor
•Periodic re-abstraction throughout the project, i.e., every few months or a few times per year
•Reviewing re-abstraction results, i.e., discrepancies or difficulty areas, with abstractors
•Measurement of inter- or intrarater reliability, i.e., a measure of agreement between two abstractions
•Monitoring abstractor performance, i.e., through re-abstraction, manual review, or computerized data checks
•Data quality control activities, e.g., re-abstraction, manual review, or computerized data checks, should be ongoing for the duration of the project rather than occur just once during the study or registry
•Clinical review of abstracted cases, i.e., a clinician or senior abstractor looking through the abstracted data screen by screen or page by page
•Visual review, i.e., a person looking through the abstracted data screen by screen or page by page, of abstracted cases
Sharing quality control information with participating facilities
•Using re-abstracted data to improve institutional data accuracy, i.e., as feedback to the healthcare facility medical records or data quality program
•External audits of abstraction at local facilities
•Returning data discrepancies identified during re-abstraction or other data quality control activities to local facilities for correction
•Providing data quality reports to sites
•Reporting of data quality by facility

^*^ Opposite valence factors, “Lack of abstractor training decreases accuracy of abstracted data,” “An incomplete review of the medical record (e.g., not reading all pages from the required time period) decreases the accuracy of abstracted data,” “Data element definitions that lack suggestions for where in the chart to find data values,” “Data abstracted from a complete medical record are more accurate than those that are abstracted from medical records with omissions,” “Abstractor (human) error is a factor in decreasing the accuracy of abstracted data,” and “Data abstracted from a medical record that is free from error are more accurate than those abstracted from a medical record containing errors,” were omitted from framework.

^†^ Combined factors “Misuse of the coding system” and “Misunderstanding the coding system,” and moved to the training category.

^‡^ Original text “Abstractor human error” restated to create an actionable item.

^§^ “Data elements requiring the abstractor to do calculations (e.g., convert units or score questionnaires) are less accurate than those that do not” and “Data elements that are abstracted directly from medical records) are more accurate than those requiring mapping or interpretation” were combined.

At the highest level, the framework provides four areas where a priori activities to ensure data accuracy should be considered: 1) choice of data source within the medical record, 2) abstraction methods and tools, 3) abstraction environment, and 4) abstraction human resources.

Further, we describe abstraction as a system—a system conceptualization requires feedback as an ongoing activity throughout the abstraction for a study or project. Based on the factors reported in the literature and verified through the Delphi, the feedback most often will consist of re-abstraction to identify discrepancies, reporting those discrepancies to the abstractors, and making changes to the controllable factors to decrease the discrepancy rate. Thus, the re-abstraction feedback is a quantitative indicator of data accuracy used to control the error rate. At the highest level, the framework describes two essential mechanisms to achieve the desired data accuracy from MRA. The first mechanism can be thought of as quality assurance—for example, prospective training, procedures, and job aids to ensure adequate accuracy. The second mechanism is a quality control action in which the accuracy is measured and used to guide adjustments to the abstraction process, tools, and human resources.

### 3.6 Framework Evaluation

In a manner similar to translation and back-translation, we tested the framework for coverage of factors reported by the current literature. The framework (Table 5) was tested against the recent literature from 2011 and 2012. The 66 articles in the 2011–2012 test set were reviewed to identify factors affecting the accuracy of abstracted data. The 222 identified factors were matched to the factors in the framework. The comparison yielded 208 semantic matches and 14 factors that did not match any of the 80 factors in the framework (94% coverage). The 14 non-matches aligned well under the higher level categories (13 to abstraction methods and tools and one to human resources). All statements in the literature of quality assurance or control activities were accounted for by the framework. The 14 mentions coded to high-level categories were not added to the framework and remain an area for future inquiry.

In addition to assessment of coverage, the framework was applied to the 36 articles from the test set reporting studies based in whole or in part on data abstracted from medical records to assess reporting of MRA quality assurance or control in each of the four categories. Articles mentioning one or more factors from the framework were scored as positive for the high-level category in which the factor resided. Table 6 shows the percentage of articles reporting methodology across the four high-level categories and quality control methods. The test set exhibits significant underreporting of important aspects of MRA methodology (i.e., the source from which the data were obtained, how the data were collected, and what if any quality control was performed). Perhaps most importantly, only three of the 36 clinical studies made quantitative report of the discrepancy rate (i.e., inter- or intra-rater reliability of re-abstraction).

**Table 6:** Frequency of reporting MRA methods.

	Clinical studies (36)	Non-clinical studies^*^ (30)
1) Data source within the medical record	0 (0%)	0 (0%)
2) Abstraction methods and tools	18 (50%)	22 (73%)
3) Abstraction environment	0 (0%)	0 (0%)
4) Abstraction human resources	15 (42%)	19 (63%)

Values are presented as number of studies reporting (%).

^*^ Category includes validation of administrative data, performance measures, or indicators (18); data quality assessment (11); and questionnaire validation (1).

## Discussion

### 4.1 Validity of Factors Reported in the Literature

The low percentage of new factors added by the Delphi indicates that the literature-reported factors have sufficient completeness upon which to build a model of factors affecting accuracy of data abstracted from medical records. Further, the consistency between the factors identified in Delphi Round 1 and those identified in our literature review indicates agreement between the literature and perceptions of expert abstractors. The majority of the items had high means and low variance—that is, most items reported as important in the literature were confirmed by the Delphi groups’ ratings being reliable and with low variability. Given the restricted range—that is, starting with factors already reported as important in the literature—this result confirms that the two Delphi groups largely agreed that the top factors reported in the literature were perceived as affecting the accuracy of data abstracted from medical records.

### 4.2 Factors Refuted by the Delphis

The two strongly refuted factors, “necessity of the RN credential” and “blinding of abstractors,” were contentious in the literature. Some argued for necessity of the RN credential due to the associated knowledge of data flow and documentation in the healthcare environment, ability to locate information in the medical record, and fluency in medical language.[5,18–21] The opposing argument was that individuals with clinical knowledge were more apt to interpret information in the medical record rather than rigidly follow abstraction guidelines. Participants having the RN credential strongly correlated with the perceived necessity of the credential.

The literature was similarly conflicted regarding blinding of abstractors. Some argued that blinding abstractors to study aims or endpoints helped prevent bias.[7,20,22] Others argued that full knowledge of the study purpose and endpoints was necessary for abstractors to do a good job.[21,23,24]

The low rating for presence of multiple diagnoses or procedures is puzzling. The presence of multiple diagnoses or procedures in medical records was cited multiple times in the literature as a factor influencing the accuracy of abstracted data. Two large and robust studies in abstraction for billing conducted in 1977 by the Institute of Medicine reported this as a major finding.[25,26] The reported difficulty was assigning a primary diagnosis or complaint from multiple possible problems. It is possible that this is no longer a factor, or that while it may be a significant factor in claims abstracting, it is not problematic in clinical research, registries, or QI. The refuting of centralized abstraction by the registry/QI Delphi participants is puzzling—a 1989 study of central versus local abstraction in oncology clinical trials concluded that centralized abstractors were consistently more often correct and that central abstraction should be considered an acceptable alternative to local abstraction in the context of regional programs.[27] However, the findings could be relevant only in oncology, in clinical trials, may no longer be valid, or the perceptions of the abstractors may be incorrect. The 1977 and 1989 studies were the only reported studies testing factors impacting accuracy in MRA.[25–27]

### 4.3 Comparison with an Existing Framework

With one recent exception,[23] the MRA literature is empirical and not based in theory. Engel et al. present a model they call the Medical Record Review Conduction Model.[23] The model depicts two roles (a study investigator and an abstractor), three things (an abstraction manual, data sources, and abstraction tools), and one activity (data quality analysis), with lines representing interactions between the roles, things, and action. The interactions are not further characterized. The model was published as a perspective article, and the presented model was not systematically derived or evidence-based. Although the model prompts readers to utilize an abstraction manual and an abstraction tool, it is not clear from the model what other factors may affect the accuracy of abstracted data and how that impact may occur. We significantly expanded this work by synthesizing and assessing content validity of a framework of factors affecting data accuracy in MRA.

### 4.4 Implications of the Framework

Based on the low number of factors mentioned in any single included article, we did not expect to uncover such a large number of factors in the literature or from Round 1 of the Delphi. The high number of factors impacting data accuracy in MRA demonstrates the complexity and multifaceted nature of MRA. The potential subjectivity of the task and the varying extent to which the subjectivity has been constrained (i.e., through tools, processes, and review) may explain the high and highly variable discrepancy rates associated with MRA. The large number of factors and variably constrained subjectivity means that the needed level of data accuracy for a study will not likely be achieved or maintained solely through prospective interventions; it is likely that some source of error not initially considered will occur. Thus, ongoing assessment to detect unanticipated discrepancies is needed throughout the abstraction process. For example, periodic re-abstraction performed on a representative sample of cases that provides a discrepancy rate (inter- or intra-rater reliability) where discrepancies are analyzed and a root cause assigned, ultimately providing feedback to improve the affected aspects of the abstraction process. This underscores the need for both prospective quality assurance and ongoing monitoring and control.

### 4.5 Underreporting MRA Methodology and Quality

Underreporting of MRA methodology could not be confirmed prior to the existence of a comprehensive conceptual model of the factors that affect data accuracy in MRA. The underreporting of MRA methodology in the literature is partially understandable given the lack of available information about MRA methodology; researchers likely planned and conducted such studies based on personal or local ideas and experience about which factors of MRA impacted data accuracy. Investigators and research teams were likely unaware of the entirety of factors that affect data accuracy, which of them may be important for their study, and how to prevent, mitigate and control them.

However, if even a portion of what was not documented was not done, many studies based on abstracted data have been based on data of unknown quality. Since measures of data accuracy are not required to be reported along with study results, the capability of the data to support the conclusions drawn cannot be assessed by the reader. In the context of high average discrepancy rates reported in the literature for MRA,[2] the lack of methodology and data quality reporting is a serious omission from MRA-based studies. In a recent systematic literature review and pooled analysis, MRA was associated with the highest and most variable discrepancy rate of four evaluated data collection and processing methods.[2] The results here provide one explanation why data error rates in MRA may be so large and highly variable: the large number of factors affecting data accuracy in MRA and the lack of generalized knowledge about them.

In MRA, failure at any one factor can undermine the ability of abstracted data to support conclusions drawn. As such, reports of research results in the literature should in some way document abstraction quality assurance and control methodology as well as report a measured discrepancy rate. Although space limitations in journals will not permit full description of quality assurance and control of abstraction processes, a statement of high-level categories covered along with the average discrepancy rate would be encouraging. Even more encouraging would be addition of the abstraction procedures and guidelines in supplemental material.

### 4.6 Application of the Framework

To ensure quality in MRA, researchers and research teams can rely on the four high-level categories of the framework as a guide for covering the major aspects of MRA. Researchers and research teams can also use the 80 low-level framework factors (Table 5) as examples of factors impacting accuracy of data abstracted from medical records and select from them those applicable to a particular study. The factor list is not complete; a complete list is likely unobtainable. Factors that may affect MRA may vary across clinical specialties and other aspects including data origination and processing. Thus, these results should be considered with respect to a particular study or project. We emphasize that no MRA strategy is complete without a mechanism for control.

Our ultimate concern is for the dependability and reproducibility of research results based on MRA. Consistent and predictable data accuracy can only be achieved when researchers are able to survey their data collection situation, identify threats to data accuracy, and apply appropriate error prevention, mitigation, and control methods. The framework developed and validated here can be directly used by researchers and their teams in this way (e.g., as a checklist in planning MRA-based studies).

## Limitations

The literature review and test set were restricted to articles in the English language; additional factors may have been reported in non-English articles. Homogeneity of participants is a critical factor in the Delphi process. Our Delphi participants were homogenous with respect to abstraction setting (clinical research vs. registry/QI) and experience level, but there is significant variation of practice within each setting. The higher variability seen in the registry and QI Delphi may have been a result of such heterogeneity of the participants. Additionally, more than 200 of the factors mentioned in the literature (i.e., the bottom 74%, or those factors with three or fewer mentions in the articles included in the literature review) could not be evaluated. Thus, the model, while likely useful, is incomplete with respect to the universe of factors that may potentially affect the quality of abstracted data. For this reason, and based on the evaluation, we organized the factors in a hierarchical model with exhaustiveness and mutual exclusivity of factors at the top level.

Handling of concepts such as combining or splitting based on semantic similarity, dissimilarity, or equivalence is dependent on the required conceptual granularity. The goal of this research was to inform quality assurance and control activities in MRA processes; thus, we based the desired level of granularity on aspects of the abstraction process that researchers can either assess or control. Our concept handling is therefore colored by the intended application of this research. For transparency and reproducibility, we have delineated all concept handling decisions from the literature review to the final framework in S3 Appendix. The original data files are available upon request for anyone who wishes to further explore the topic under different frameworks and for different applications.

## Further Research

The factors identified in the literature but not assessed in the Delphi process remain a topic for further evaluation. Other areas for further research include testing use of the framework as an intervention to improve accuracy of data abstracted from medical records, and observational monitoring of the literature reporting results based on abstracted data to track methodological reporting.

## Conclusions

Prior to this work, MRA was largely conducted using inconsistent methods and without evidence-based methodology. The framework generated through this research directly addresses this situation. The large number and breadth of factors identified through this work demonstrates the need for such a framework, while infrequent methodological reporting in the literature underscores it. Based on the content validity Delphi processes and subsequent evaluation against the recent literature, we conclude that the factors mentioned in the literature are active in practice today. From the consistency between the two Delphi processes, we conclude that the factors affecting accuracy are generalizable across practice settings (e.g., clinical research, registries, and QI projects).

From the number of factors and the high level of agreement between expert abstractors, we conclude that data accuracy in MRA is a complex, many-faceted problem. Thus, solutions for improving, controlling, and ensuring accuracy of abstracted data will necessarily be multi-faceted. Ultimately, a priori definition of methods, tools, and resources alone is necessary but insufficient to achieve and demonstrate that data are capable of supporting conclusions drawn from them. The abstraction discrepancy rate should be measured, monitored throughout the abstraction process, used as feedback to control the process, and reported with research results.

## Supporting Information

S1 Checklist PRISMA Checklist. (DOC)

S1 Appendix PubMed search strategy. (DOC)

S2 Appendix Articles included in the review. (DOC)

S3 Appendix Concept handling from literature review to the final framework. (DOC)

S4 Appendix Participant statements regarding factors. (DOC)

S1 Fig Concept handling steps and resulting number of factors (see S3 Appendix for description of decisions leading to addition or removal of factors). (TIFF)
