# Supplementary material for: Factors Affecting Accuracy of Data Abstracted from Medical Records
Source: PLoS One. 2015 Oct 20;10(10):e0138649. doi: 10.1371/journal.pone.0138649 (PMC4615628; doi:10.1371/journal.pone.0138649)
Supplement: S1 Checklist — (DOC) [file pone.0138649.s001.doc]

| **Section/topic** | **#** | **Checklist item** | **Reported on page #** |
| --- | --- | --- | --- |
| **TITLE** | | |  |
| Title | 1 | Identify the report as a systematic review, meta-analysis, or both.  This was not done because the systematic review was done to provide factors mentioned in the text of relevant articles that were later validated (content validity) in a two-group Delphi process. The factors were used to create a framework, and then the framework was assessed against the recent literature. Thus, the systematic review was input rather than the topic of the paper. If the editors or reviewers feel differently, I will reconsider. | NA  See comment |
| **ABSTRACT** | | |  |
| Structured summary | 2 | Provide a structured summary including, as applicable: background; objectives; data sources; study eligibility criteria, participants, and interventions; study appraisal and synthesis methods; results; limitations; conclusions and implications of key findings; systematic review registration number. | 2 |
| **INTRODUCTION** | | |  |
| Rationale | 3 | Describe the rationale for the review in the context of what is already known. | 3-4 |
| Objectives | 4 | Provide an explicit statement of questions being addressed with reference to participants, interventions, comparisons, outcomes, and study design (PICOS).  The systematic review was of articles describing data quality of the medical record abstraction / chart review process. This review was NOT for the purpose of synthesizing evidence from human subjects research, i.e., no participants, no interventions, no comparisons, thus, PICOS does not apply.  The statement of questions addressed is provided in the objective of the structured abstract. | 2 statement of questions |
| **METHODS** | | |  |
| Protocol and registration | 5 | Indicate if a review protocol exists, if and where it can be accessed (e.g., Web address), and, if available, provide registration information including registration number. | NA |
| Eligibility criteria | 6 | Specify study characteristics (e.g., PICOS, length of follow-up) and report characteristics (e.g., years considered, language, publication status) used as criteria for eligibility, giving rationale. | 5 |
| Information sources | 7 | Describe all information sources (e.g., databases with dates of coverage, contact with study authors to identify additional studies) in the search and date last searched. | 5 |
| Search | 8 | Present full electronic search strategy for at least one database, including any limits used, such that it could be repeated. | Appendix S1 |
| Study selection | 9 | State the process for selecting studies (i.e., screening, eligibility, included in systematic review, and, if applicable, included in the meta-analysis). | 5 |
| Data collection process | 10 | Describe method of data extraction from reports (e.g., piloted forms, independently, in duplicate) and any processes for obtaining and confirming data from investigators. | 6 |
| Data items | 11 | List and define all variables for which data were sought (e.g., PICOS, funding sources) and any assumptions and simplifications made. | 6 |
| Risk of bias in individual studies | 12 | Describe methods used for assessing risk of bias of individual studies (including specification of whether this was done at the study or outcome level), and how this information is to be used in any data synthesis.  We are not reviewing study results. Instead, we are looking for possible factors that impact the accuracy of the medical record abstraction process. | NA |
| Summary measures | 13 | State the principal summary measures (e.g., risk ratio, difference in means).  The systematic review was conducted to identify factors mentioned in the articles as impacting the accuracy of the medical record abstraction process. There were no summary measures other than the total number of mentions, and the number of distinct factors. | Pg 11 lines 238-241 |
| Synthesis of results | 14 | Describe the methods of handling data and combining results of studies, if done, including measures of consistency (e.g., I^2^) for each meta-analysis.  Results of studies or synthesis of such were not a part of this research. Data handling is described in the Methods section for each part of the work (systematic literature review, Assessing content validity via Delphi processes, The framework, and evaluating the framework) on pgs 5-11 | 5-11 |

Page 1 of 2

| **Section/topic** | **#** | **Checklist item** | **Reported on page #** |
| --- | --- | --- | --- |
| Risk of bias across studies | 15 | Specify any assessment of risk of bias that may affect the cumulative evidence (e.g., publication bias, selective reporting within studies).  See comment for item 12 | 22 lines 473-474 |
| Additional analyses | 16 | Describe methods of additional analyses (e.g., sensitivity or subgroup analyses, meta-regression), if done, indicating which were pre-specified.  Interclass Correlation Coefficient (ICC) analysis of the Delphi responses to identify factors with low reliability was conducted and reported on page 16. | 16 |
| **RESULTS** | | |  |
| Study selection | 17 | Give numbers of studies screened, assessed for eligibility, and included in the review, with reasons for exclusions at each stage, ideally with a flow diagram. | Text pg 5 and Figure 1 |
| Study characteristics | 18 | For each study, present characteristics for which data were extracted (e.g., study size, PICOS, follow-up period) and provide the citations. | 6 |
| Risk of bias within studies | 19 | Present data on risk of bias of each study and, if available, any outcome level assessment (see item 12). | NA |
| Results of individual studies | 20 | For all outcomes considered (benefits or harms), present, for each study: (a) simple summary data for each intervention group (b) effect estimates and confidence intervals, ideally with a forest plot. | NA |
| Synthesis of results | 21 | Present results of each meta-analysis done, including confidence intervals and measures of consistency. | NA |
| Risk of bias across studies | 22 | Present results of any assessment of risk of bias across studies (see Item 15). | NA |
| Additional analysis | 23 | Give results of additional analyses, if done (e.g., sensitivity or subgroup analyses, meta-regression [see Item 16]). | NA |
| **DISCUSSION** | | |  |
| Summary of evidence | 24 | Summarize the main findings including the strength of evidence for each main outcome; consider their relevance to key groups (e.g., healthcare providers, users, and policy makers). | NA |
| Limitations | 25 | Discuss limitations at study and outcome level (e.g., risk of bias), and at review-level (e.g., incomplete retrieval of identified research, reporting bias). | NA |
| Conclusions | 26 | Provide a general interpretation of the results in the context of other evidence, and implications for future research. | 19-21, 22-23 |
| **FUNDING** | | |  |
| Funding | 27 | Describe sources of funding for the systematic review and other support (e.g., supply of data); role of funders for the systematic review.  The project received support from Grants UL1RR024128 and UL1RR024148 to Duke University and the University of Texas Health Science Center Houston, respectively, from the National Center for Research Resources (NCRR), and from Grant 5K99LM011128 from the National Library of Medicine (NLM). Both NCRR and NLM are components of the National Institutes of Health (NIH). The contents of this publication are solely the responsibility of the authors and do not necessarily represent the official view of the NIH. |  |

*From:*  Moher D, Liberati A, Tetzlaff J, Altman DG, The PRISMA Group (2009). Preferred Reporting Items for Systematic Reviews and Meta-Analyses: The PRISMA Statement. PLoS Med 6(6): e1000097. doi:10.1371/journal.pmed1000097

For more information, visit: **www.prisma-statement.org**.

Page 2 of 2
