# Supplementary material for: Factors Affecting Accuracy of Data Abstracted from Medical Records
Source: PLoS One. 2015 Oct 20;10(10):e0138649. doi: 10.1371/journal.pone.0138649 (PMC4615628; doi:10.1371/journal.pone.0138649)
Supplement: S1 Appendix — (DOC) [file pone.0138649.s002.doc]

**S1 Appendix**

**PubMed Search Strategy**

(((abstraction[Title/Abstract]) OR ("chart review" [Title/Abstract])) OR ("medical record review"[Title/Abstract])) AND ("clinical trial"[Title/Abstract] OR registry[Title/Abstract] OR "clinical research"[Title/Abstract] OR quality[Title/Abstract] OR "performance"[Title/Abstract]) AND (error[Title/Abstract] OR accuracy[Title/Abstract] OR "data quality"[Title/Abstract] OR errors[Title/Abstract] OR decision[Title/Abstract] OR reliability[Title/Abstract] OR validity[Title/Abstract])
