# Supplementary material for: Factors Affecting Accuracy of Data Abstracted from Medical Records
Source: PLoS One. 2015 Oct 20;10(10):e0138649. doi: 10.1371/journal.pone.0138649 (PMC4615628; doi:10.1371/journal.pone.0138649)
Supplement: S2 Appendix — (DOC) [file pone.0138649.s003.doc]

**S2 Appendix**

**Articles Included in the Review**

1. Feinstein AR, Pritchett JA, Schimpff CR. The epidemiology of cancer therapy. 3. The management of imperfect data. *Arch Intern Med* 1969;123(4):448-61.

2. Feinstein AR, Pritchett JA, Schimpff CR. The epidemiology of cancer therapy. IV. The extraction of data from medical records. *Arch Intern Med* 1969;123(5):571-90.

3. Berkanovic E. An appraisal of Medicaid records as a data source. *Med Care* 1974;12(7):590-5.

4. Institute of Medicine. *Reliability of Hospital Discharge Abstracts.* Washington DC: National Academy of Sciences 1977.

5. Institute of Medicine. *Reliability of Medicare Hospital Discharge Records.* Washington DC: National Academy of Sciences 1977.

6. Demlo LK, Campbell PM, Brown SS. Reliability of information abstracted from patients' medical records. *Med Care* 1978;16(12):995-1005.

7. Gardiner RC. Quality considerations in medical records abstracting systems. *J Med* *Syst* 1978;2(1):31-43.

8. Haley RW, Schaberg DR, McClish OK, et al. The accuracy of retrospective chart review in measuring nosocomial infection rates. Results of validation studies in pilot hospitals. *Am J Epidemiol* 1980;111(5):516-33.

9. Herrmann N, Cayten CG, Senior J, et al. Interobserver and intraobserver reliability in the collection of emergency medical services data. *Health Serv Res* 1980;15(2):127-43.

10. Bertelsen J. Who should abstract medical records? A study of accuracy and cost. *Eval Health Prof* 1981;4(1):79-92.

11. Birnbaum D, King LA. Disadvantages of infection surveillance by medical record chart review. *Am J Infect Control* 1981;9(1):15-7.

12. Brown F, Major problems in data accuracy remain unsolved. *Top Health Rec Manage* 1982;2(4):4-8.

13. Johnson OG. Data: let the user beware. *Top Health Rec Manage* 1982;2(4):8-12.

14. Meads S, Cooney JP. The medical record as a data source: use and abuse. *Top Health Rec Manage* 1982;2(4):23-32.

15. Horwitz RI, Yu EC. Assessing the reliability of epidemiologic data obtained from medical records. *J Chronic Dis* 1984;37(11):825-31.

16a. Kosecoff J, Brook RH, Chassin MR, et al. *Medical Record Abstraction Forms for Assessing Compliance With NIH Consensus Conference Recommendations.* Santa Monica, CA: Rand Corporation 1987.

16b. Kahn KL, Chassin MR, Rubenstein LV, et al. *Medical Record Abstraction Form and Guidelines for Assessing Quality of Care for Hospitalized Patients With Congestive Heart Failure.* Santa Monica, CA: Rand Corporation 1988.

16c. Kosecoff J, Rubenstein LV, Kahn KL, et al. *Medical Record Abstraction Form and Guidelines for Assessing Quality of Care for Hospitalized Patients With Acute Myocardial Infarction.* Santa Monica, CA: Rand Corporation 1988.

16d. Roth CP, Kahn KL, Sherwood MJ, et al. *Medical Record Abstraction Form and Guidelines for Assessing Quality of Care for Hospitalized Patients With Pneumonia*. Santa Monica, CA: Rand Corporation 1988.

16e. Sherwood MJ, Kahn KL, Kosecoff J, et al. *Medical Record Abstraction Form and Guidelines for Assessing Quality of Care for Hospitalized Patients With Hip Fracture.* Santa Monica, CA: Rand Corporation 1988.

16f. Rubenstein LV, Kosecoff J, Kahn KL, et al. *Medical Record Abstraction Form and Guidelines for Assessing Quality of Care for Hospitalized Patients With Cerebrovascular Accident.* Santa Monica, CA: Rand Corporation 1988.

17. Crede WB, Hierholzer WJ Jr. Mortality rates as a quality indicator: a simple answer to a complex question. *Infect Control Hosp Epidemiol* 1988; 9(7):330-2.

18. Findley TW, Daum MC. Research in physical medicine and rehabilitation III: The chart review or how to use clinical data for exploratory retrospective studies. *Am J Phys Med* 1989;68(3):150–157.

19. Jasperse OM, Ahmed SW. The Mid-Atlantic Oncology Program’s comparison of two data collection methods. *Control Clin Trials* 1989;10(3):282-9.

20. Larks M. Panel confirms data uniformity is key to improving healthcare quality. *Patient Acc* 1989;12(6):2-3.

21. Law M, Ryan B, Townsend E, et al. Criteria mapping: a method of quality assurance. *Am J Occup Ther* 1989;43(2):104-9.

22. Williamson JW, Fehlauer CS, Gaiennie J, et al. Assessing quality of ambulatory care: a comparative analysis in rural versus tertiary general medical clinics. *Qual Assur Util Rev* 1991;6(1):8-15.

23. Beard CM, Yunginger JW, Reed CE, et al. Interobserver variability in medical record review: an epidemiological study of asthma. *J Clin Epidemiol* 1992;45(9):1013-20.

24. Muse A, Smith PF, Mikl J. The quality of computerized hospital discharge data. *Am J Public Health* 1992;82(7):1044.

25. Audet AM, Scott HD. The Uniform Clinical Data Set: an evaluation of the proposed national database for Medicare’s quality review program. *Ann Inter Med* 1993;119(12):1209-13.

26. Burney RE, Gies ME, Williams D, et al. A trial of structured implicit review of randomly selected peer review organization cases. *Clin Perform Qual Health Care* 1993;1(4):214-8.

27. Rozewski CM. A method for measuring and reporting manual data extraction reliability. *Comput Methods Programs Biomed* 1993;41(1):17-31.

28. Aaronson LS, Burman ME. Use of health records in research: reliability and validity issues. *Res Nurs Health* 1994;17(1):67-73.

29. Korman HJ, Sirls LT, Kirkemo AK. Success rate of modified Pereyra bladder neck suspension determined by outcomes analysis. *J Urol* 1994;152(5 Pt. 1):1453-7.

30. Kuntoro, LaPorte RE, Mazumdar S. Approaches to quality control with an application to a new cancer registry in a developing country. *J Clin Epidemiol* 1994;47(7):779-86.

31. Nettleman MD, Nelson AP. Adverse occurrences during hospitalization on a general medicine service. *Clin Perform Qual Health Care* 1994;2(2):67-72.

32. Pollock BH. Quality assurance for interventions in clinical trials: multicenter data monitoring, data management, and analysis. *Cancer* 1994;74(9 Suppl):2647-52.

33. Ellerbeck EF. In reply to comments by Localio AR, Landis JR. Quality of chart review for quality of care. *JAMA* 1995;274(20):1585-6.

34. Mullins RJ, Veum-Stone J, Hedges JR, et al. An analysis of Hospital Discharge Index as a trauma data base. *J Trauma* 1995;39(5):941-8.

35. Sapien RE, Olson LM, Horne-Lucero LC, et al. Retrospective versus concurrent review on the quality of care of pediatric trauma patients. *Pediatr Emerg Care* 1995;11(3):162-6.

36. VonKoss Krawchuk H, Moore ML, Richardson L. Using health care records as sources of data for research. *J Nurs Meas* 1995;3(1):3-12.

37. Wyatt J. Acquisition and use of clinical data for audit and research. *J Eval Clin Pract* 1995;1(1):15-27.

38. Audet AM, Goodnough LT, Parvin CA. Evaluating the appropriateness of red blood cell transfusions: the limitations of retrospective medical record reviews. *Int J Qual Health Care* 1996;8(1):41-9.

39. Gibbs D. For debate: 250th anniversary of source document verification. *BMJ* 1996;313:798.

40. Gilbert EH, Lowenstein SR, Kozioi-Mclain J, et al. Chart reviews in emergency medicine research: Where are the methods? *Ann Emerg Med* 1996;27(3):305-8.

41. Hopkins A, Irwin P, Wallace H. Conceptual differences amongst the data collection instruments used in clinical audit. *J Eval Clin Pract* 1996;2(2):153-6.

42. Smith AJ. Chart reviews made simple. *Nurs Manage* 1996;27(8):33-4.

43. Schwartz RJ, Panacek EA. Basics of research (Part 7): Archival data research. *Air Med J* 1996;15(3):119-24.

44. Brown JM, Haining SA, Hale JM. Views on local data management in cancer clinical trials. *Clin Oncol* *(R Coll Radiol)* 1997;9(6):403-6.

45. Dresser MV, Feingold L, Rosenkranz SL, et al. Clinical quality measurement: comparing chart review and automated methodologies. *Med Care* 1997;35(6):539-52.

46. Forbes SA, Duncan PW, Zimmerman MK. Review criteria for stroke rehabilitation outcomes. *Arch Phys Med Rehabil* 1997;78(10):1112-6.

47. Harris HE, Ellison GT, Holliday M, et al. Methodological considerations in the design of an obstetric database abstracted from medical records. *Methods Inf Med* 1997;36(3):191-200.

48. Huff ED. Comprehensive reliability assessment and comparison of quality indicators and their components. *J Clin Epidemiol* 1997;50(12):1395-404.

49. Wu L, Ashton CM. Chart review: a need for reappraisal. *Eval Health Prof* 1997;20(2):146-63.

50. Banks NJ. Designing medical record abstraction forms. *Int J Qual Health Care* 1998;10(2):163-7.

51. Use a streamlined approach for medical record abstraction. *Qual Lett Healthc Lead* 1998;10(6):14-5.

52. Stange KC, Zyzanski SJ, Smith TF, et al. How valid are medical records and patient questionnaires for physician profiling and health services research? A comparison with direct observation of patients visits. *Med Care* 1998;36(6):851-67.

53. Buckley NA, Whyte IM, Dawson AH, et al. Preformatted admission charts for poisoning admissions facilitate clinical assessment and research. *Ann Emerg Med* 1999;34(4):476-82.

54. Every NR, Frederick PD, Robinson M, et al. A comparison of the national registry of myocardial infarction 2 with the cooperative cardiovascular project. *J Am Coll Cardiol* 1999;33(7):1886-94.

55. Griffiths R, Hindle D. The effectiveness of AN-DRGs in classification of acute admitted patients with diabetes. *Health Inf Manag* 1999;29(2):77-83.

56. Kasner SE, Chalela JA, Luciano JM, et al. Reliability and validity of estimating the NIH stroke scale score from medical records. *Stroke* 1999;30(8):1534-7.

57. Lorenzoni L, Da Cas R, Aparo UL. The quality of abstracting medical information from the medical record: the impact of training programmes. *Int J Qual Health Care* 1999;11(3):209-13.

58. McEvoy P. Using patients’ records as a source of data. *Nurs Stand* 1999;13(36):33-6.

59. Owen JL, Bolenbaucher RM, Moore ML. Trauma registry databases: a comparison of data abstraction, interpretation, and entry at two level I trauma centers. *J Trauma* 1999;46(6):1100-4.

60. Schuyl ML, Engel T. A review of the source document verification process in clinical trials. *Drug Info Journal* 1999;33:789-97.

61. Allison JJ, Wall TC, Spettell CM, et al. The art and science of chart review. *Jt* *Comm J Qual Improv* 2000;26(3):115-36.

62. Aronsky D, Haug PJ. Assessing the quality of clinical data in a computer-based record for calculating the pneumonia severity index. *J Am Med Inform Assoc* 2000;7(1):55-65.

63. Gandhi TK, Seger DL, Bates OW. Identifying drug safety issues: from research to practice. *Int J Qual Health Care* 2000;12(1):69-76.

64. Humphries KH, Rankin JM, Carere RG, et al. Co-morbidity data in outcomes research: are clinical data derived from administrative databases a reliable alternative to chart review? *J Clin Epidemiol* 2000;53(4):343-9.

65. Hunt JP, Cherr GS, Hunter C, et al. Accuracy of administrative data in trauma: splenic injuries as an example. *J Trauma* 2000;49(4):679-86.

66. Katz RI, Lagasse RS. Factors influencing the reporting of adverse perioperative outcomes to a quality management program. *Anesth Analg* 2000;90(2):344-50.

67. Luck J, Peabody JW, Dresselhaus TR, et al. How well does chart abstraction measure quality? A prospective comparison of standardized patients with the medical record. *Am J Med* 2000;108(8):642-9.

68. Howell KM. Beyond EDITS: using computer in checking melanoma staging. *J Registry Manag* 2000;27(3):105-8.

69. Mismash ML. Case summary: a key to data quality. *J Registry Manag* 2000;27(2):60-63.

70. Peabody JW, Luck J, Glassman P, et al. Comparison of vignettes, standardized patients, and chart abstraction: a prospective validation study of 3 methods for measuring quality. *JAMA* 2000;283(13):1715-22.

71. Surawicz TS, McCarthy BJ, Jukich PJ, et al. The accuracy and completeness of primary brain and central nervous system tumor data: results from the Central Brain Tumor Registry of the United States. *J Registry Manag* 2000;27(2):51-55.

72. Abbott KC, Bohen EM, Welch PG, et al. Analyzing process variation in chart review using a networked spreadsheet application in the Walter Reed Army Medical Center Nephrology Clinic. *Mil Med* 2001;166(9):771-3.

73. Fritz A. The SEER Program’s commitment to data quality. *J Registry Manag* 2001;28(1):35-40.

74. Hilsenbeck SG. Quality in cancer registries—where have we been and where are we going? *J Registry Manag* 2001;28(1):22-23.

75. Kung HC, Hanzlick R, Spitler JF. Abstracting data from medical examiner/coroner reports: concordance among abstractors and implications for data reporting. *J Forensic Sci* 2001;46(5):1126-31.

76. Brewster DH, Stockton D, Harvey J, et al. Reliability of cancer registration data in Scotland, 1997. *Eur J Cancer* 2002;38(3):414-7.

77. Cassidy LD, Marsh GM, Holleran MK, et al. Methodology to improve data quality from chart review in the managed care setting. *Am J Manag Care* 2002;8(9):787-93.

78. Cook SF, Visscher WA, Hobbs CL, et al. Project IMPACT: results from a pilot validity study of a new observational database. *Crit Care Med* 2002;30(12):2765-70.

79. Kerr EA, Smith DM, Hogan MM, et al. Comparing clinical automated, medical record, and hybrid data sources for diabetes quality measures. *Jt Comm J Qual Improv* 2002;28(10):555-65.

80. Simmons B, Bennett F, Nelson A, et al. Data abstraction: designing the tools, recruiting and training the data abstractors. *SCI Nurs* 2002;19(1):22-4.

81. Geiger AM, Greene SM, Pardee RE 3rd, et al. A computerized system to facilitate medical record abstraction in cancer research (United States). *Cancer Causes Control* 2003;14(5):469-76.

82. Reisch LM, Fosse JS, Beverly K, et al. Training, quality assurance, and assessment of medical record abstraction in a multisite study. *Am J Epidemiol* 2003;157(6):546-51.

83. Robertson J. Cardiovascular point of care initiative: enhancements in clinical data management. *Qual Manag Health Care* 2003;12(2):115-21.

84. Scherer R, Zhu Q, Langenberg P, et al. Comparison of information obtained by operative note abstraction with that recorded on a standardized data collection form. *Surgery* 2003;133(3):324-30.

85. Watt A, Williams S, Leek K, et al. Keen eye on core measures: Joint Commission data quality study offers insights into data collection, abstracting processes. *J AHIMA* 2003;4(10):20-5.

86. Drebing C, Movitz R, Lyon P, et al. Documenting pathways to dementia care: relative validity of questionnaire, interview, and medical record formats. *Am J Alzheimers Dis Other Demen* 2004;19(3):187-97.

87. Downey L, Lafferty WE, Tao G, et al. Evaluating the quality of sexual health care provided to adolescents in Medicaid managed care: a comparison of two data sources. *Am J Med Qual* 2004;19(1):2-11.

88. Haan CK, Adams M, Cook R. Improving the quality of data in your database: lessons from a cardiovascular center. *Jt* *Comm J Qual Saf* 2004;30(12):681-8.

89. Hellings P. A rich source of clinical research data: medical records and telephone logs. *J* *Pediatr Health Care* 2004;18(3):154-5.

90. Hess DR. Retrospective studies and chart reviews. *Respir Care* 2004;49(10):1171-4.

91. Hofer TP, Asch SM, Hayward RA, et al. Profiling quality of care: is there a role for peer review? *BMC Health Serv Res* 2004;4(1):9.

92. Mansson J, Nilsson G, Bjorkelund C, et al. Collection and retrieval of structured clinical data from electronic patient records in general practice: a first-phase study to create a health care database for research and quality assessment. *Scand* *J Prim Health Care* 2004;22(1):6-10.

93. Mitchell S, Bartoli H. Re-abstraction studies to assess data quality for use in the development of a grouping methodology. *2004 IFHRO Congress & AHIMA Convention Proceedings*. http:// library.ahima.org (accessed February 27, 2014).

94. Mullooly J, Drew L, DeStefano F, et al. Quality assessments of HMO diagnosis databases used to monitor childhood vaccine safety. *Method Inf Med* 2004;43(2):163-70.

95. Soroka M, Feldman L, Crump T. Quality-of-care review of optometric records: inter-rater reliability. *J Healthc Qual* 2004;26(5):29-33.

96. Takayanagi R, Watanabe K, Nakahara A, et al. Items of concern associated with source document verification of clinical trials for new drugs. *Yakugaku Zasshi* 2004;124(2):89-92.

97. Worster A, Haines T. Advanced statistics: understanding medical record review (MRR) studies. *Acad Emerg Med* 2004;11(2):187-92.

98. Badcock D, Kelly AM, Kerr D, et al. The quality of medical record review studies in the international emergency medicine literature. *Ann Emerg Med* 2005;45(4):444-7.

99. Barnato AE, Labor RE, Freeborne NE, et al. Qualitative analysis of Medicare claims in the last 3 years of life: a pilot study. *J Am Geriatr Soc* 2005;53(1):66-73.

100. Benin AL, Vitkauskas G, Thornquist E, et al. Validity of using an electronic medical record for assessing quality of care in an outpatient setting. *Med Care* 2005;43(7):691-8.

101. Birman-Deych E, Waterman AD, Yan Y, et al. Accuracy of ICD-9-CM codes for identifying cardiovascular and stroke risk factors. *Med Care* 2005;43(5):480-5.

102. Booth FV, Short M, Shorr AF, et al. Application of a population-based severity scoring system to individual patients results in frequent misclassification. *Crit Care* 2005;5;9(5):R522-9.

103. Eder C, Fullerton J, Benroth R, et al. Pragmatic strategies that enhance the reliability of data abstracted from medical records. *Appl Nursing Res* 2005;18(1):50-4.

104. Jansen AC, van Aalst-Cohen ES, Hutten BA, et al. Guidelines were developed for data collection from medical records for use in retrospective analyses. *J Clin Epidemiol* 2005;58(3):269-74.

105. Lowenstein SR. Medical record reviews in emergency medicine: the blessing and the curse. *Ann Emerg Med* 2005;45(4):452-5.

106. McKenzie K, Walker S, Besenyei A, et al. Assessing the concordance of trauma registry data and hospital records. *HIM J* 2005;34(1):3-7.

107. Nagurney JT, Brown OF, Sane S, et al. The accuracy and completeness of data collected by prospective and retrospective methods. *Acad Emerg Med* 2005;12(9):884-95.

108. Pan L, Fergusson D, Schweitzer I, et al. Ensuring high accuracy of data abstracted from patient charts: the use of a standardized medical record as a training tool. *J Clin Epidemiol* 2005;58(9):918-23.

109. Roos LL, Gupta S, Soodeen RA, et al. Data quality in an information-rich environment: Canada as an example. *Can J Aging* 2005;24(Suppl 1):153-70.

110. Worster A, Bledsoe RD, Cleve P, et al. Reassessing the methods of medical record review studies in emergency medicine research. *Ann Emerg Med* 2005;45(4):448-51.

111. Yawn BP, Wollan P. Interrater reliability: completing the methods description in medical records review studies. *Am J Epidemiol* 2005;161(10):974-7.

112. Breslauer C. Could 100% source document verification become a risk in a fixed unit price environment? *Monitor* 2006;43-7.

113. Du XL, Key CR, Dickie L, et al. Information on chemotherapy and hormone therapy from tumor registry had moderate agreement with chart reviews. *J Clin Epidemiol* 2006;59(1):53-60.

114. Gearing RE, Mian LA, Barber J, et al. A methodology for conducting retrospective chart review research in child and adolescent psychiatry. *J Can Acad Child Adolesc Psychiatry* 2006;15(3):126-34.

115. Grunfeld E, Lethbridge L, Dewar R, et al. Towards using administrative databases to measure population-based indicators of quality of end-of-life care: testing the methodology. *Palliat Med* 2006;20(8):769-77.

116. Lienard JL, Quinaux E, Fabre-Guillevin E, et al. Impact of on-site initiation visits on patient recruitment and data quality in a randomized trial of adjuvant chemotherapy for breast cancer. *Clin Trials* 2006;3(5):486-92.

117. McDermott MF, Lenhardt RO, Catrambone CD, et al. Adequacy of medical chart review to characterize emergency care for asthma: findings from the Illinois Emergency Department Asthma Collaborative. *Acad Emerg Med* 2006;13(3):345-8.

118. Persell SD, Wright JM, Thompson JA, et al. Assessing the validity of national quality measures for coronary artery disease using an electronic health record. *Arch Intern Med* 2006;166(20):2272-7.

119. Soran A, Nesbitt L, Mamounas EP, et al. Centralized medical monitoring in phase Ill clinical trials: the National Surgical Adjuvant Breast and Bowel Project (NSABP) experience. *Clin Trials* 2006;3(5):478-85.

120. Williams SC, Watt A, Schmaltz SP, et al. Assessing the reliability of standardized performance indicators. *Int J Qual Health Care* 2006;18(3):246-55.

121. Williams GW. The other side of clinical trial monitoring; assuring data quality and procedural adherence. *Clin Trials* 2006;3(6):530-7.

122. Zissiadis Y, Harper E, Harper C. Development of a clinical chart audit programme. *Australas Radiol* 2006;50(4):349-54.

123. Abdelhak M, Grostick S, Hanken MA, et al., eds. *Health Information: Management of a Strategic Resource*, Third Edition. St. Louis, MO: Elsevier 2007: Chapter 14.

124. Alley LG, Chen VW, Wike JM, et al. CDC-NPCR's Breast, Colon, and Prostate Cancer Data Quality and Patterns of Care study: overview and methodology. *J Registry Manag* 2007;34(4):148-57.

125. Gliklich RE, Dreyer NA, eds. *Registries for Evaluating Patient Outcomes: A User's Guide.* AHRQ Publication No. 07-EHC001-1. Rockville, MD: Agency for Healthcare Research and Quality 2007.

126. Hemmila MR, Jakubus JL, Wahl WL, et al. Detecting the blind spot: complications in the trauma registry and trauma quality improvement. *Surgery* 200;142(4):439-48.

127. Lash TL, Fox MP, Thwin SS, et al. Using probabilistic corrections to account for abstractor agreement in medical record reviews. *Am J Epidemiol* 2007;165(12):1454-61.

128. Pawlson LG, Scholle SH, Powers A. Comparison of administrative-only versus administrative plus chart review data for reporting HEDIS hybrid measures. *Am J Manag Care* 2007;13(10):553-8.

129. Solomon DH, Stedman M, Licari A, et al. Agreement between patient report and medical record review for medications used for rheumatoid arthritis: the accuracy of self-reported medication information in patient registries. *Arthritis Rheum* 2007;57(2):234-9.

130. Thoburn KK, German RR, Lewis M, et al. Case completeness and data accuracy in the Centers for Disease Control and Prevention’s National Program of Cancer Registries. *Cancer* 2007;109(8):1607-16.

131. Williams CA, Mosley-Williams AD, Overhage JM. Arthritis quality indicators for the Veterans Administration: implications for electronic data collection, storage format, quality assessment, and clinical decision support. *AMIA Annu Symp Proc* 2007:806-10.

132. Connelly LM. Retrospective chart reviews. *Medsurg Nurs* 2008;17(5):322-3.

133. Cunningham R, Sarfati D, Hill S, et al. An audit of colon cancer data on the New Zealand Cancer Registry. *N Z Med J* 2008;121(1279):46-56.

134. Flood M, Small R. Researching labour and birth events using health information records: methodological challenges. *Midwifery* 2009;25(6):701-10.

135. Floerchinger-Franks G, Carson S. Implementing a statewide trauma registry: recommendations from the Idaho Trauma Registry Pilot Project. *J Registry Manag* 2008;35(3):109-12.

136. German RR, Wike JM, Bauer KR, et al. Quality of cancer registry data: Findings from CDC- NPCR’s Breast and Prostate Cancer Data Quality and Patterns of Care Study. *J Registry Manag* 2008;35(2):67-74.

137. Kunac DL, Reith DM. Preventable medication-related events in hospitalised children in New Zealand. *N Z Med J* 2008;121(1272):17-32.

138. Pakhomov S, Bjornsen S, Hanson P, et al. Quality performance measurement using the text of electronic medical records. *Med Decis Making* 2008;28(4):462-70.

139. Reeves MJ, Mullard AJ, Wehner S. Inter-rater reliability of data elements from a prototype of the Paul Coverdell National Acute Stroke Registry. *BMC Neurol* 2008;8:19.

140. Seng JS, Mugisha E, Miller JM. Reliability of a perinatal outcomes measure: the Optimality Index-US. *J Midwifery Women's Health* 2008;53(2):110-4.

141. To T, Estrabillo E, Wang C, et al. Examining intra-rater and inter-rater response agreement: a medical chart abstraction study of a community-based asthma care program. *BMC Med Res Methodol* 2008;8:29.

142. Bray F, Parkin DM. Evaluation of data quality in the cancer registry: principles and methods. Part 1: comparability, validity and timeliness. *Eur J Cancer* 2009;45(5):747-55.

143. Engel L, Henderson C, Fergenbaum J, et al. Medical record review conduction model for improving interrater reliability of abstracting medical-related information. *Eval Health Prof* 2009;32(3):281-98.

144. Hanauer DA, Englesbe MJ, Cowan JA Jr., et al. Informatics and the American College of Surgeons National Surgical Quality Improvement Program: automated processes could replace manual record review. *J Am Coll Surg* 2009;208(1):37-41.

145. National Committee for Quality Assurance. *HEDIS 2009, Appendix 2 - HEDIS Roadmap Section 4: Medical Record, Volume 5.* www.ncqa.org (accessed February 26, 2014).

146. Houston TK, Wall TC, Willet LL, et al. Can residents accurately abstract their own charts? *Acad Med* 2009;84(3):391-5.

147. Hrisos S, Eccles MP, Francis JJ, et al. Are there valid proxy measures of clinical behaviour? A systematic review. *Implement Sci* 2009;4:37.

148. Kergoat MJ, Leclerc BS, Leduc N, et al. Quality of care assessment in geriatric evaluation and management units: construction of a chart review tool for a tracer condition. *BMC Geriatr* 2009;9:34.

149. Linder JA, Kaleba EO, Kmetik KS. Using electronic health records to measure physician performance for acute conditions in primary care: empirical evaluation of the community-acquired pneumonia clinical quality measure set. *Med Care* 2009;47(2):208-16.

150. Needham DM, Sinopoli DJ, Dinglas VD, et al. Improving data quality control in quality improvement projects. *Int J Qual Health Care* 2009;21(2):145-50.
