# Supplementary material for: Factors Affecting Accuracy of Data Abstracted from Medical Records
Source: PLoS One. 2015 Oct 20;10(10):e0138649. doi: 10.1371/journal.pone.0138649 (PMC4615628; doi:10.1371/journal.pone.0138649)
Supplement: S3 Appendix — (DOC) [file pone.0138649.s004.doc]

**S3 Appendix**

**Concept Handling from Literature Review to the Final Framework**

Handling of concepts such as combining or splitting based on semantic similarity, dissimilarity, or equivalence is dependent on the required conceptual granularity. The goal of this research was to inform quality assurance and control activities in MRA processes; thus, we based the desired level of granularity on aspects of the abstraction process that researchers can either assess or control. Our concept handling is therefore colored by the intended application of this research. For this reason, we have delineated all concept handling decisions from the literature review to the final framework in this appendix. The original data files are available upon request for anyone who wishes to further explore the topic under different frameworks and for different applications.

The codes with which the factor statements from the literature and Delphi Round 1 were coded were developed through a first-pass review of the 155 included articles. First pass review generated an initial set of 309 codes. These codes were entered into NVivo qualitative analysis software (QSR International, Victoria, Australia) for use during the double independent review and coding of the 155 included articles and, later, the Delphi Round 1 results.

Double independent review and coding of the 155 included articles provided 2385 individual statements of factors impacting the accuracy of abstracted data. Double independent coding of these factors stated in the literature resulted in 292 unique factors. Seventeen of the original 309 codes were ultimately not used or combined with others during the coding and adjudication process to resolve coding differences between the two coders.

Round 1 of the Delphi returned 227 statements of factors. Six of the 227 items fell outside of the working definition of MRA, and five items could not be classified due to ambiguity of the information provided by the participant, leaving 216 mentions. Of these 216, 92 distinct factors were identified.

For Round 2, the Round 1 factors were combined with the top 75 literature factors. Combining the 75 top literature factors and the 25 top Delphi Round 1 factors provided 100 total factors, 89 of them distinct (Table 3). Only these top mentioned factors could be carried forward in Round 2 because the participants were consented for a 1-hour or shorter time commitment per Delphi round.

The 89 distinct factors as stated in the Delphi Round 1 and coded from the literature were sorted into 14 categories arising from the data. In preparation for creation of the Round 2 questionnaire, each category was reviewed for exhaustive and mutually exclusive coverage. Each factor was reviewed for singularity of concept, consistent granularity, and definitional precision. This review resulted in the combination of eight factors with others because the level of detail required to differentiate each was inconsistent with the other factors and was so detailed that the authors felt it was quite possible that no real differentiation was intended in the original statements from the Delphi Round 1 or literature. This lumping resulted in 81 distinct factors. One factor, “Misuse or misunderstanding of the coding system,” was split into two, bringing the total to 82 factors. A second factor, “Training manual,” was split into five, which brought the total to 86 distinct factors. A third factor, “…practice exercises with feedback…,” was split into two factors, bringing the total to 87 factors. A fourth factor, “data elements requiring judgment…,” was retained but split, adding four types of subjective data elements, bringing the total to 91 factors. A fifth factor, “well designed data collection form…,” was retained but split, adding two additional factors representing evidence-based form design principles from the literature, bringing the total to 93 factors. Six additional factors were added to make five categories more complete, bringing the total to 99 factors carried forward into Delphi Round 2.

In the interclass correlation analysis, three factors had an overall rating lower than neutral (Table 4); all of these were rated between mildly disagree and neutral. The registry and QI Delphi rated seven factors lower than neutral (Table 4). The clinical research Delphi rated two factors lower than neutral. All but one factor rated lower than neutral originated from the literature. In summary, the factors exhibiting low reliability (Table 4) dropped an additional four factors from the list. Factors were dropped if they exhibited low reliability or importance from either Delphi—a total of 11 (11%) out of the 99 Delphi-vetted factors were not upheld by the Delphi. Thus, the Delphi process resulted in 88 verified factors.

Six factors of opposite valence made it through to the Delphi-vetted set of 88 factors. With respect to adding factors to the framework, three options for handling these were available: 1) add opposite valence to all factors for completeness, 2) drop the opposite valence factors, or 3) leave the six opposite valence factors in the framework. Ultimately, we felt that the opposite valence factors would cause confusion in the framework. We had no evidence that valence mattered for the other 82 factors, and we had no evidence that statements of one direction or the other—for example, “lack of training decreases the accuracy of data abstracted from medical records” versus “training abstractors increases the accuracy of data abstracted from medical records”—were intentional. We did not have additional time in the interview Delphi rounds to explore this; we ultimately chose to drop the six opposite valence factors (as noted in Table 5). Thus, the framework does not answer the question “If doing something increases accuracy, does lack of that something decrease accuracy?” The 82 factors were further consolidated during framework development by removing and lumping two sets of factors with closely related factors (noted in main article Table 5). The framework contains 80 factors.

The 204 (292 – 88) untested factors were not included in the framework. Factors were grouped into the initial systems theory-based four high-level categories: 1) the medical record, 2) abstraction methods and tools, 3) abstraction environment, and 4) abstraction human resources.
