# Supplementary material for: Factors Affecting Accuracy of Data Abstracted from Medical Records
Source: PLoS One. 2015 Oct 20;10(10):e0138649. doi: 10.1371/journal.pone.0138649 (PMC4615628; doi:10.1371/journal.pone.0138649)
Supplement: S4 Appendix — (DOC) [file pone.0138649.s005.doc]

**S4 Appendix**

**Participant Statements Regarding Factors**

**RN Credential**

“I don’t think education is a factor at all; there are health education and health information people who could do just as good a job. Having a healthcare background is the important thing.”

“The Nurses are familiar with the chart tab order, with the physician’s handwriting and medical terminology and abbreviations.”

“I’m not an RN and a lot of our study coordinators are not RNs. Can obtain the knowledge base without the credentials by experience.”

“RNs are better at gleaning the material requested from the information [in the medical record]. They better understand the diagnosis and dynamics and what to look for.”

“Someone with a clinical background will look at things in more detail.”

“It doesn’t matter what their credentials are; if they have had the same project specific training/education, there shouldn't be any difference.”

“I don't have an RN and I can abstract.”

“I am not an RN but I’ve gotten knowledge from just working in the field and having physicians explain things to me.”

“I am an RN, so I am partial. A nurse’s curve of knowledge, medical background and medical terminology makes a difference.”

“There are other degrees besides RN that help.”

“An abstractor doesn’t have to have RN credentials. Having a medical background and experience is more important.”

“I am not a nurse. Abstracting does not require an RN, it requires training.”

“Nurses have better comprehension of medical terms and clinical matters. They have a better understanding of the study and diagnoses because of their background and education.”

**Blinding Abstractors to Study Aims**

“Sometimes the abstractors can have a better idea of where to find data if they understand what they are supposed to be looking for and not working in the dark.”

“Even if the abstractor didn’t know the aim of the study, if they are a good abstractor, their accuracy would still be the same.”

“I think they need to know study aims. This question makes more sense for a clinical researcher than for a registry person.”

“If abstractors know the goal of the study it could bias the abstractor’s interpretation.”

“Blinding is really not important.”

“Blinding abstractors decreases accuracy. Understanding the aims or purpose helps the abstractor to know the intent of the question in the context of the study.”

“There is so much information in medical records – so many different angles you can look at. If the abstractor does not have a focus, it can affect the information they are abstracting.”

“I had to know what I was looking for in abstracting data. You need to know the purpose.”

“Disagree because you are just pulling data points.”

“It would help the abstractor to know what the purpose of the study is.”

**Centralized Abstraction**

“In the real world, copies are illegible and unreadable; someone gets their thumb in the way or pages are missing.”

“If abstraction is done locally, you can ask questions without having to send questions back and forth.”

“We are all electronic at our hospital so it is easy to set up access for another user electronically by remote if we have a business agreement with them.”

“No errors in a photocopied chart – apples to apples, but if going from paper to electronic entry would increase the possibility of error.”

“It does not matter where the data are abstracted; can be central or dispersed.”

“Having the same people doing the abstraction for a study is helpful because there is more consistency and accuracy between cases.”

“Using one coding center does not necessarily increase accuracy. Can achieve accuracy with dispersed sites.”

“Based on my experience with centralized abstraction, you don't always get what you want. Centralized personnel may not dig as deep for information.”

**High Study/Project Complexity**

“If the abstractors are competent and given the time to do it right, then there is no problem.”

“I am perplexed by people who disagree mildly. Abstracting something that is more complex could cause more errors or inaccurate data.”

“There are different issues with multiple facets different specialty physicians dictate differently; they also use different methods.”

“If an abstractor is trained properly, they are able to abstract accurately whether it was complex or not.”

“If the study is complex, the questions are confusing or the form is long, the questions could be misunderstood. Difficult studies, very detailed studies and studies on unusual topics can also lead to decreased accuracy.”

“As long as the abstraction guidelines and the instructions are well written, complexity should not affect abstraction accuracy. The documentation in the guidelines affects the accuracy.”

“Because you have a tendency to spend a lot of time looking for all the information and making sure it is correct. Complex registries require more quality time.”

“The more complex, the more difficult it is to pull out all of the details.”

“Whether there is a lot to look for or a little to look for, your eyes are trained.”

“No matter how complex the study or registry, once you train, accuracy should be better.”

“The more complex the data, the more chance of making errors—e.g., when abstracting histology for oncology studies, it helps to be an RN or have a medical background.”

**High Volume of Data in Medical Records**

“Only if you have lazy abstractors.”

“The more information, the longer you are looking at one big document, the greater the chance of error.”

“I treat them all the same whether the medical record is thick or thin.”

“Thick medical records have more information and more inconsistent information.”

“The more entries, the more complex the patient. Many entries for the patient means more time to go through the patient’s record.”

“The abstractor may miss things in a thick record.”

“I don't feel that it should. Because I am very detail oriented, I read everything and I pull out all of the data.”

“I don't see the correlation at all.”

“Abstractor fatigue makes the abstractor more likely to miss something when going through a long medical record.”

“The shorter records, the least amount of data that I need to collect. Thicker records contain more information.”

“Volume does not have anything to do with accuracy.”

“This is not so much of an issue with an EMR. The thicker the medical record, the more information I have available to find things. A short chart does not always include everything needed.”

“A thicker medical record denotes more available information and more opportunity to find what you are looking for.”

**Care Provided by Multiple Providers/Facilities**

“Not if you are taking the time [to look at all the documentation of the multiple providers at the multiple facilities]. It is possible to get more information from multiple facilities.”

“It depends on the training. Patients are usually seen by more than one provider, so it should not affect the abstraction accuracy.”

“Information from multiple facilities would be harder to abstract.”

“It depends on the competency of the abstractor. There may be differences in the documentation but the abstraction should not be less accurate.”

“It makes it more difficult but not more accurate. An abstractor that is trained well can abstract from multiple facility records and providers accurately. It comes down to experience.”

“It should not make any difference in accuracy, if documents from different facilities are being abstracted. It doesn’t matter where the documents come from.”

“I am assuming they are looking for similar things, so there would be more of the same data elements.”

“Not everyone documents in the same way. Documentation from multiple providers may be inconsistent from one provider to the next, even if they work in the same department. Also, the charts of patients from multiple facilities or who have been seen by multiple physicians are more complex.”

“Multiple providers or multiple hospitals have no effect on accuracy of abstraction. As long as you have a good rapport with them.”

“I agree because will not see the whole picture, if care is provided at a variety of places.”

“I took that to mean multiple providers at multiple facilities, which is not so much of a factor as how the information is gathered.”

**Presence of Multiple Diagnoses/Procedures**

“If you are not paying attention.”

“If you know where to look and what to look for, you can abstract the data.”

“Different physicians, different specialists, different disciplines use different terminology for diagnoses. More complex patients have more entries and accuracy can be decreases.”

“I don’t know if it would make a difference.”

“Whoever does the documentation, you would hope it would be documented accurately.”

“The abstractor should be looking more at the complex diagnoses and procedures. The more complex the more you are looking.”

“Multiple diagnoses can decrease accuracy because you are so busy getting all of the diagnosis data.”

“More complex cases make accuracy less and the medical record longer. Reviewer fatigue after looking at one record for a long time.”

**Abstractors With Different Levels of Experience**

“Accuracy decreases as the accuracy of the individual abstractor. If you are doing a difficult study with a high degree of precision and new abstractors, you will not get very good results.”

“While experience is important, there are abstractors with less experience (length of time they have been abstracting), there could be new abstractors who still have a high level of quality.”

“Experience is very important because you know what you are looking for and where to find it.”

“It is important for each abstractor to have the same amount of experience; if they have more than two years of experience they pay attention to more details.”

“It decreases accuracy when there are different levels of ability to apply knowledge based information (use knowledge in context).”

“If a study or registry is well designed with good QA measures, it should account for the differences between the skills of the abstractors.”

“If you have the same amount of training on the project, different levels of experience should not matter.”

“Sometimes new abstractors have a better understanding, and sometimes old abstractors get into bad habits.”

“Different levels of abstraction experience should not decrease the accuracy of abstraction. Abstractors should have an idea of where to find the data.”

“Previous experience makes an abstractor faster but not more accurate. We use students to do a lot of the abstracting and then have an experienced abstractor go behind them and check their work.”

“As long as they know what they are doing and what they should be looking for, it is not necessary for them to have a lot of experience.”

“It depends on the actual project and the actual data that you want to obtain. Having abstractors with different backgrounds (i.e., nurse, lab person, or an expert in the field) can bring a balance that makes the data better.”

“Abstractors with different experience levels have different perceptions. Being less familiar would decrease the accuracy.”

“One person is consistent; more than one person is inconsistent. The more hands in the pie the messier it can get.”

**Abstracting From Narrative Text**

“Even if the information is in scanned progress notes and dictation, if the guidelines are clear, you can find it.”

“The abstractor may miss some things buried in a narrative. Forms may serve as prompts to promote completeness.”

“The abstractor should be able to find it regardless of where or in what form it resides in the medical record.”

“As long as the narrative text has been created accurately by the physician the data can be abstracted as accurately from the narrative text. Sure, forms are easier to abstract, but not more accurate.”

“I don't know if it would detract from accuracy.”

**Coding Data While Abstracting**

“You can code and abstract simultaneously. I don’t see where it would confuse anyone. If abstracting you have a clearer picture of the whole case, after having coded it.”

“This is what we do in our facility—abstract and code at the same time page by page. We run reports from the abstracted data.”

“Coding at the same time as getting the data speeds up the process and makes it more accurate.”

“Coders need to have more consistency; first get the written diagnosis then add the code, then enter the code.”

“Coding data is an element of abstracting. It just adds another step but does not necessarily decrease accuracy.”

“It depends on what is being coded. If you have information that helps you choose the correct code, it helps you choose the correct code. Need to have all of the information to make the coding decisions.”

“Currently, I am coding data while abstracting and it does not affect my accuracy, but I have been doing this for 4 years on the same project.”

“I’ve done that many times and it did not make a difference, even when I was a coder.”

“If a good experienced abstractor, as long as she knows where to look for the code list, it shouldn't matter if it is on the data collection form or in a manual.”

“Is very subjective.”

**Same Information Found in Multiple Places**

“It should increase accuracy—there are more places to find the information. If there is conflicting information, you go with the preponderance.”

“Information is often found in multiple places and sometimes the terms are different but they mean the same thing.”

“Accuracy is decreased because you have to look in more than one spot for a piece of information and you may miss one of the spots.”

“When patients with many procedures and diagnoses are recorded in different locations, interpretation is left open and results and data may not be captured.”

“The abstractor may not review the entire record and then just chooses what they could easily find in the chart.”

“Most often information found in different places in the chart confirms information from another source or information found in another place in the chart. If inconsistencies, we would abstract every possibility documented in the chart. We have options for conflicting information.”

“Recording information in multiple chart locations just gives the abstractor more places to find the information.”

“Our EMR dumps things from the original source into other documents—but because it is an EMR, and copying from the original source, the information is the same.”

**Overall**

“One of the problems with surveys is that many of the answers are not black or white but have many variables that could make the answer strongly disagree or relatively strongly agree for the same question. In other words, “it depends” are the two words that I found myself repeating in my head as I answered the questions. For instance, review of data quality results will increase or decrease abstracting results—it depends! We all know that just because you give providers their outcomes it does not guarantee that they change their practice behaviors. Then of course, this type of free text answer does not allow for easy reporting, but without it the results may not represent the whole picture.”

“I assume that accuracy of data includes identification of all cases that meet criteria for a study.”

“Individual skills, experience, and concentration are going to vary from day to day and will have an effect on the information.”
