# Supplementary figures and images for: Factors Affecting Accuracy of Data Abstracted from Medical Records
Source: PLoS One. 2015 Oct 20;10(10):e0138649. doi: 10.1371/journal.pone.0138649 (PMC4615628; doi:10.1371/journal.pone.0138649)

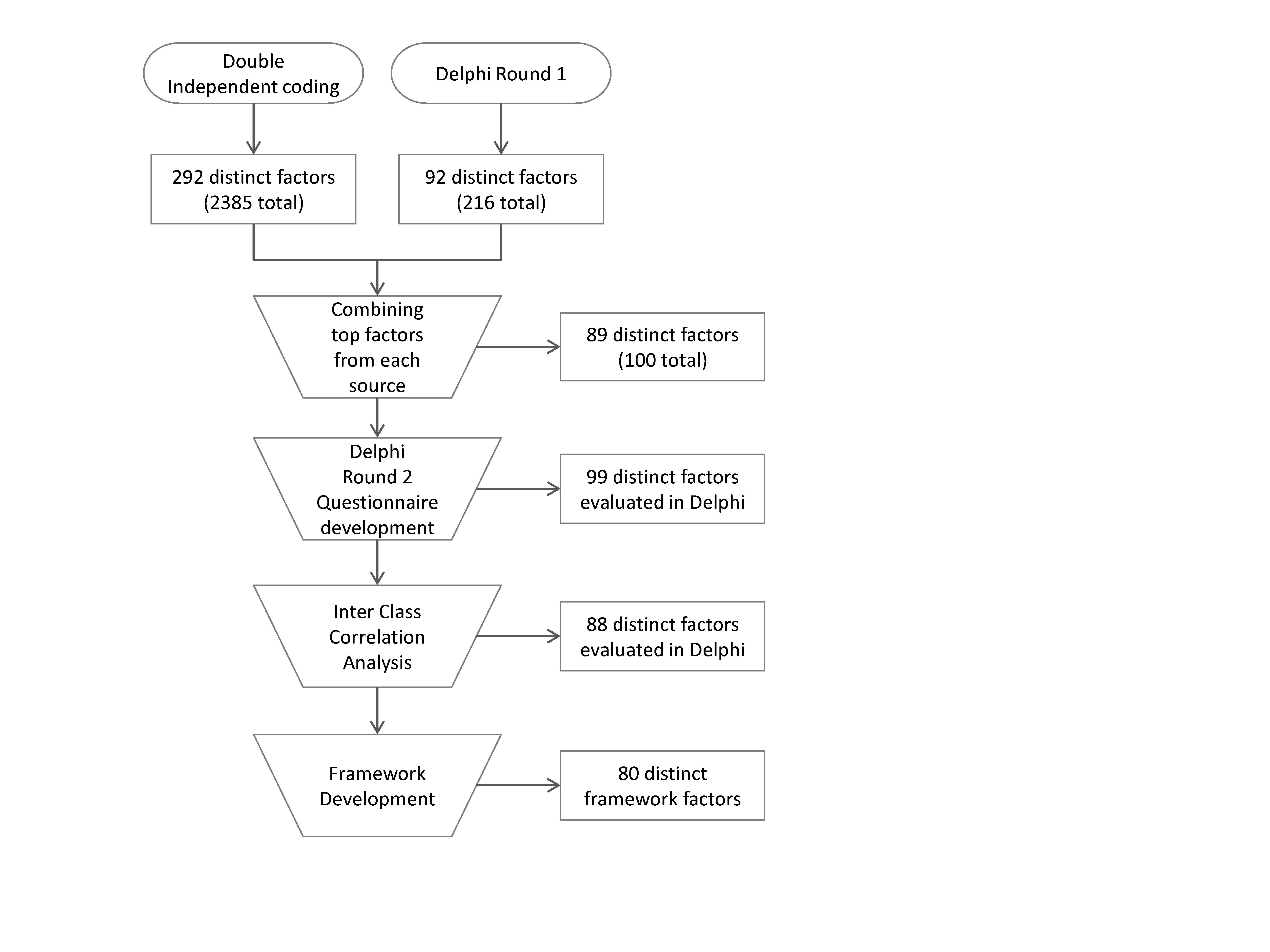

Supplement: S1 Fig — (TIFF) [file pone.0138649.s006.tiff]
